# A comparative analysis of the burden, trends and inequalities of tracheal, bronchus, and lung cancer in India from 2000 to 2021: A systematic analysis for the Global Burden of Disease study 2021

**DOI:** 10.1371/journal.pone.0322646

**Published:** 2025-05-07

**Authors:** Shuting Rao, Haijiang Wu, Guibin Zhang, Wenli Dong, Luzhe Cui, Yashu Wang, Xinna Deng

**Affiliations:** 1 Department of Oncology, Hebei Medical University, Shijiazhuang, Hebei, China; 2 Department of Oncology, Hebei General Hospital, Shijiazhuang, Hebei, China; 3 Department of Pathology, Hebei Medical University, Shijiazhuang, Hebei, China; 4 Department 1 of Thoracic Surgery, Hebei General Hospital, Shijiazhuang, Hebei, China; 5 Department of Radiation Oncology, Hebei North University, Zhangjiakou, Hebei, China; 6 Department of Radiation Oncology, North China University of Science and Technology, Tangshan, Hebei, China; Christian Medical College, INDIA

## Abstract

**Background:**

Understanding the tracheal, bronchus, and lung (TBL) cancer burden caused by tobacco exposure in India can help local governments implement targeted measures for prevention and treatment of the disease.

**Methods:**

The burden of TBL cancer deaths and disability-adjusted life years (DALYs) attributable to tobacco exposure from 2000 to 2021 were presented by age, sex, and region. A Joinpoint model was used to analyze temporal trends of the disease, while decomposition analysis was conducted to quantify the contributions of population growth, aging, and epidemiological changes. In addition, the age-period-cohort (APC) model was implemented to assess the effects of age, period, and cohort on tobacco-related TBL cancer deaths and DALYs burden. Finally, age-standardized deaths and DALYs rates for TBL cancer attributable to tobacco exposure were projected through 2035.

**Results:**

In 2021, Mizoram recorded the highest age-standardized rates of TBL cancer deaths and DALYs attributable to tobacco exposure, regardless of sex. Uttar Pradesh and West Bengal consistently exhibited the highest number of deaths and DALYs associated with tobacco exposure across the three age groups analyzed. Population growth and aging are the primary drivers behind the increasing burden of TBL cancer. Overall, the risk of tobacco-related lung cancer death increased with age. There are differential period and cohort effects between male and female populations. In the future, the increase in age-standardized rates of deaths and DALYs attributable to secondhand smoke exposure will be more pronounced among males.

**Conclusion:**

Despite ongoing efforts to control the tobacco epidemic, the burden of TBL cancer related to tobacco remains high in India. Each state in India should adopt targeted measures based on local conditions to address the health threats posed by tobacco.

## Introduction

Tobacco is recognized as a significant risk factor for global public health. Every year, more than 8 million people lose their lives due to tobacco exposure, including approximately 1.3 million non-smokers who are exposed to secondhand smoke [1]. Tobacco is widely recognized as a crucial risk factor for cancer, particularly tracheal, bronchus, and lung cancer, and remains the leading contributor to cancer-related deaths and DALYs burden [2]. Smoking and secondhand smoke are important sources of tobacco exposure, and both are causally associated with lung cancer development [3,4]. Studies have shown that the risk of death from lung cancer among smokers is about 20 times higher than that among never-smokers. Additionally, exposure to secondhand smoke both at home and in the workplace is significantly associated with an increased risk of lung cancer [5,6]. Secondhand smoke may raise the overall risk of cancer in never-smokers, especially lung and breast cancer, with a more pronounced effect on women [7]. As the world’s second-largest tobacco consumer, India currently has a tobacco use prevalence rate of 32.8%, contributing to roughly one-sixth of tobacco-related deaths worldwide each year [8,9]. Given the substantial tobacco consumption and the significant mortality burden associated with it, it is imperative to conduct a thorough assessment of the tobacco-related lung cancer burden in India. A comprehensive study examined the burden of various cancers in India from 1990 to 2016, while Sharma et al. specifically explored the cancer burden attributable to tobacco exposure from 1990 to 2019 [10,11]. Additionally, a recent study investigated the burden of tracheal, bronchus, and lung cancer across 66 Silk Road countries, including India [12]. However, none of these studies have provided a thorough or detailed analysis of tobacco-related tracheal, bronchus, and lung cancer at the national level in India. Therefore, this study aimed to analyze the sex-specific burden of tracheal, bronchus, and lung cancer deaths and disability-adjusted life years attributable to tobacco exposure in selected regions of India.

## Materials and methods

### Data source

We obtained data on TBL cancer-related deaths and DALYs burden attributable to tobacco exposure in India from 2000 to 2021 by gender, using the Global Health Data Exchange (GHDx) query tool (https://ghdx.healthdata.org/gbd-2021). To predict the future burden of tobacco-related lung cancer in India, we utilized population data from the “World Population Prospects 2024” published by the United Nations (https://population.un.org/wpp/). The tobacco exposure assessed in this study included active smoking and secondhand smoke exposure. Smoking is defined as the daily or occasional use of any smoked to bacco product. Secondhand smoke exposure refers to the current contact with tobacco smoke by non-smokers (individuals who do not smoke daily) in residential, workplace, or public settings [6,13]. One disability-adjusted life-year is equal to one year of health loss [14]. A comprehensive methodology for the Global Burden of Disease study has been published in relevant scientific publications [15–17].

### Statistical analysis

The death and DALYs burden of TBL cancer was quantified using absolute numbers, crude rates, and age-standardized rates (per 100,000 population), with a 95% uncertainty interval (UI) provided. The trend of tobacco-associated TBL cancer burden was analyzed through relative differences and the average annual percentage change (AAPC). The relative difference is calculated as the difference between the values in the two comparison years (2000 and 2021), divided by the value in 2000 and then multiplied by 100%. The average annual percent change and its 95% confidence interval (UI) over the entire study period were calculated using Joinpoint regression analysis. An increasing trend was defined as an AAPC and its 95% CI lower limit >0, while a decreasing trend was defined as an AAPC and its 95% CI upper limit <0. All other trends were classified as stable [18].

Through decomposition analysis, the contributions of population growth, age structure, and epidemiological changes to the burden of TBL cancer deaths and DALYs attributable to risk factors were assessed. According to Das Gupta’s decomposition method, DALYs related to risk factors of TBL cancer was decomposed and analyzed from age structure, population growth and epidemiological changes. The calculation formula of DALYs burden in each region is as follows:


$${DALY}_{ay,\;py,\;ey}=\sum_{i=1}^{20}\left(a_{i,\;y}*p_{y}*e_{i,\;y}\right)$$

Among them, ${DALY}_{ay,\;py,\;ey}$ represent DALY based on factors such as age structure, population and DALY rate in a specific year y. $a_{i,\;y}$ represents the proportion of population in age group i among 20 age groups in a given year y; $p_{y}$ denotes the total population in a given year y; $e_{i,\;y}$ represents the DALY rate for age category i in a given year y. The contribution of each factor to the change in DALY from 2000 to 2021 is defined by the impact resulting from changing that factor while leaving the other factors constant. For example, the effects of age structure are calculated as follows:


$$\begin{array}{cc} & [({DALY}_{a2021,\;p2000,e2000}+{DALY}_{a2021,\;p2021,\;e2021})/3+({DALY}_{a2021,\;p2000,\;e2021}+{DALY}_{a2021,\;p2021,\;e2000})/6]\\ & \;-[({DALY}_{a2000,\;p2021,e2021}+{DALY}_{a2000,\;p2000,\;e2000})/3+({DALY}_{a2000,\;p2021,\;e2000}+{DALY}_{a2000,\;p2000,\;e2021})/6] \end{array}$$

The decomposition analysis method for the burden of deaths refers to DALYs [19–23]. The age-period-cohort (APC) model was used to analyze the effects of age, period, and cohort on the burden of tobacco-related TBL cancer deaths. The age effect reflects to the impact of changes brought about by aging on the burden of disease, while the period effect captures the change in social, economic, and cultural that impact the whole population over time. The birth cohort effect refers to variations in disease burden across different birth cohorts, attributable to differences in exposure to risk factors. The age interval is typically equivalent to the period interval, requiring 5-year age groups to be paired with 5-year periods. The age range for tobacco and secondhand smoke-related TBL cancer spans from 25–29 years to 95 + years, while the age range for smoking-related TBL cancer extends from 30–34 years to 95 + years, both categorized in 5-year intervals. For the burden of risk factors within these age ranges, we selected the youngest age group as the reference, and the periods were divided into 5-year intervals from “2000-2004” to “2020-2024” with the reference period being 2000–2004. Accordingly, the analysis incorporated 19 partially overlapping 10-year birth cohorts for tobacco and secondhand smoke-related TBL cancer, ranging from 1901–1909 (median: 1905) to 1991–1999 (median: 1995). For smoking-related TBL cancer, 18 partially overlapping 10-year birth cohorts were analyzed, ranging from 1901–1909 (median: 1905) to 1986–1994 (median: 1990). The APC model outputs include longitudinal age curves, which represent the age-specific rates of the reference cohort after adjusting for period bias. These curves reflect the influence of age effects on TBL cancer trends. The relative risks for period and birth cohort are based on the age-specific rate ratios of the selected reference period and cohort, representing the influence of period and cohort effects on TBL cancer trends. The ratio (RR) indicates the value of a specific age, period, or cohort compared to the reference. An RR > 1 suggests a higher risk of disease associated with that factor compared to the reference factor. The analysis was conducted using the APC web tool provided by the National Cancer (https://analysistools.cancer.gov/apc/). The Wald chi-square test was employed to assess the statistical significance of the estimable parameters and functions, with statistical significance defined as a two-sided *P*-value < 0.05 [23–29]. The Bayesian age-period-cohort (BAPC) model was employed to predict age-standardized death and DALY rates attributable to tobacco exposure for TBL cancer up to 2035 [30,31]. In this study, we used the “BAPC” and “INLA” packages in R to perform BAPC model predictions and visualizations. All data analysis and data visualization in this study were performed using the statistical software R (version 4.2.1) and the Joinpoint Regression Analysis Software (version 4.9.1.0). This study was conducted using publicly available data from the Global Burden of Disease study and did not involve any research conducted by the authors on human or animal subjects. Therefore, ethical approval was not required.

## Results

### TBL cancer burden due to tobacco by region

The burden and trends of tobacco-related TBL cancer-related deaths among male in India are provided in S1 Table. The count of tobacco-related TBL cancer deaths increased by 84.38%, rising from 16,041 (95% UI: 13,850–18,345) in 2000 to 29,577 (95% UI: 22,853–35,625) in 2021. Among the 13 Indian states with growth rates exceeding 100%, Jharkhand recorded the lowest increase. The age-standardized mortality rate of TBL cancer attributable to tobacco was 5.19 (95% UI: 4.02–6.26), showing no significant change since 2000 (AAPC, 0.03). Mizoram, Uttarakhand, and Meghalaya showed the highest age-standardized death rates, contrasting with Jharkhand and Bihar which had the lowest. Additionally, age-standardized mortality rates declined in 11 Indian states. The number of smoking-related TBL cancer deaths increased by 83.94%, rising from 15,686 (95%UI: 3,636–17,790) in 2000 to 28,853 (95%UI: 22,386-34,673) in 2021. The age-standardized mortality rate for smoking-related TBL cancer in Andhra Pradesh has shown a decline compared to that associated with tobacco. The regions exhibiting the highest and lowest age-standardized mortality rates for smoking-associated TBL cancer were consistent with those for tobacco-related TBL cancer. The count of secondhand smoke-related TBL cancer deaths increased by 76.63%, rising from 1,789 (95%UI: 238-3,662) in 2000 to 1,013 (95%UI: 123-1,948) in 2021. A decrease in TBL cancer deaths associated with secondhand smoke was observed in Jharkhand. The age-standardized mortality rate of TBL cancer attributable to secondhand smoke was 0.31 (95% UI: 0.04–0.64) in 2021, showing a decline from 2000 (AAPC, −0.24). Furthermore, age-standardized mortality rates have decreased in 15 Indian states. The burden and trends of tobacco-related TBL cancer-related deaths among female in India are provided in S2 Table. The count of tobacco-related TBL cancer deaths increased by 140.18%, rising from 1,653 (95%UI: 1,104-2,225) in 2000 to 3,970 (95%UI: 2,641–5,653) in 2021. Among the 22 Indian states that experienced growth rates exceeding 100%, Kerala recorded the lowest increase. In 2021, the age-standardized death rate in India was 0.67(95%UI: 0.44–0.94), reflecting an increase compared to 2000 (AAPC, 0.78). Age-standardized mortality rates from tobacco-related TBL cancer have declined in 11 Indian states. Mizoram exhibited the highest age-standardized death rate, followed by Manipur and Uttarakhand, while Tamil Nadu and Jharkhand demonstrated the lowest age-standardized mortality rates. The count of smoking-related TBL cancer deaths in India was 1,269 (95%UI: 981–1619) cases, an increase of 144.43% compared to 3,101 (95%UI: 2,281–4,100) cases in 2000. The age-standardized death rate in India has increased to 0.53 (95%UI: 0.39–0.69) in 2021. Mizoram had the highest age-standardized mortality rate, followed by Manipur and Sikkim. Punjab and Tamil Nadu had the lowest age-standardized mortality rates. The count of secondhand smoke-related TBL cancer deaths increased by 119.31%, rising from 468 (95%UI: 62-895) in 2000 to 1,027 (95%UI: 146-2,117) in 2021. Among the 19 Indian states with growth rates exceeding 100%, Kerala recorded the lowest increase. The age-standardized mortality rate in India is 0.31(95%UI: 0.04–0.64) in 2021, reflecting a decline since 2000 (AAPC, −0.24). Age-standardized mortality rates declined across 8 Indian states, with the highest rates observed in Mizoram and Uttarakhand, and the lowest in Jharkhand and Bihar. Notably, the increase in tobacco-related TBL cancer deaths was not significant in Kerala, while the decline in age-standardized death rates was significant. In addition, the trends observed among men in Maharashtra were similar to those in Kerala. Furthermore, Changes in the DALYs burden of tobacco-associated TBL cancer are consistent with the trends observed for deaths, as detailed in S3 Table and S4 Table.

### Counts and crude rates of TBL cancer attributable to tobacco across different age groups

**Fig 1:**
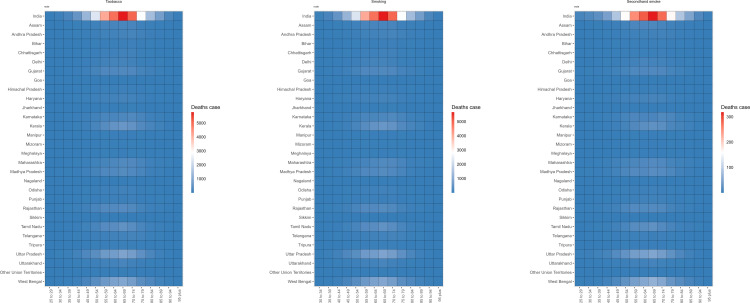
Tobacco exposure related TBL cancer deaths among male by age in different regions of India in 2021.

**Fig 2:**
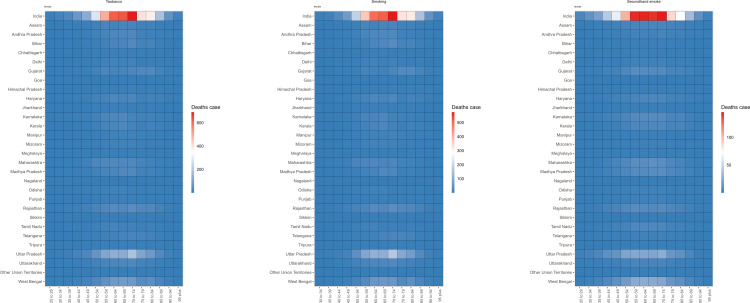
Tobacco exposure related TBL cancer deaths among female by age in different regions of India in 2021.

The age distribution of the burden of TBL cancer deaths in 2021 is shown in Figs 1 and 2 and S1-S2 Figs. The counts of TBL cancer deaths attributable to tobacco, smoking, and secondhand smoke exhibited a similar normal distribution across 8 regions: Gujarat, Kerala, Madhya Pradesh, Maharashtra, Rajasthan, Tamil Nadu, Uttar Pradesh, and West Bengal. The number of TBL cancer deaths attributable to risk factors was predominantly concentrated in the 65–69 age group. The age-specific distribution of TBL cancer death rates associated with all risk factors differed from the age distribution of death cases, as shown in S1-S2 Figs. Across most regions, the 70–74 age group exhibited the highest death rates. It is worth noting that the mortality rate among those aged over 60 in Mizoram is significantly higher than in other regions. The burden of DALYs for TBL cancer did not significantly differ from the burden of deaths (S3-S6 Figs).

### Tobacco-related TBL cancer burden in three different age groups

**Fig 3:**
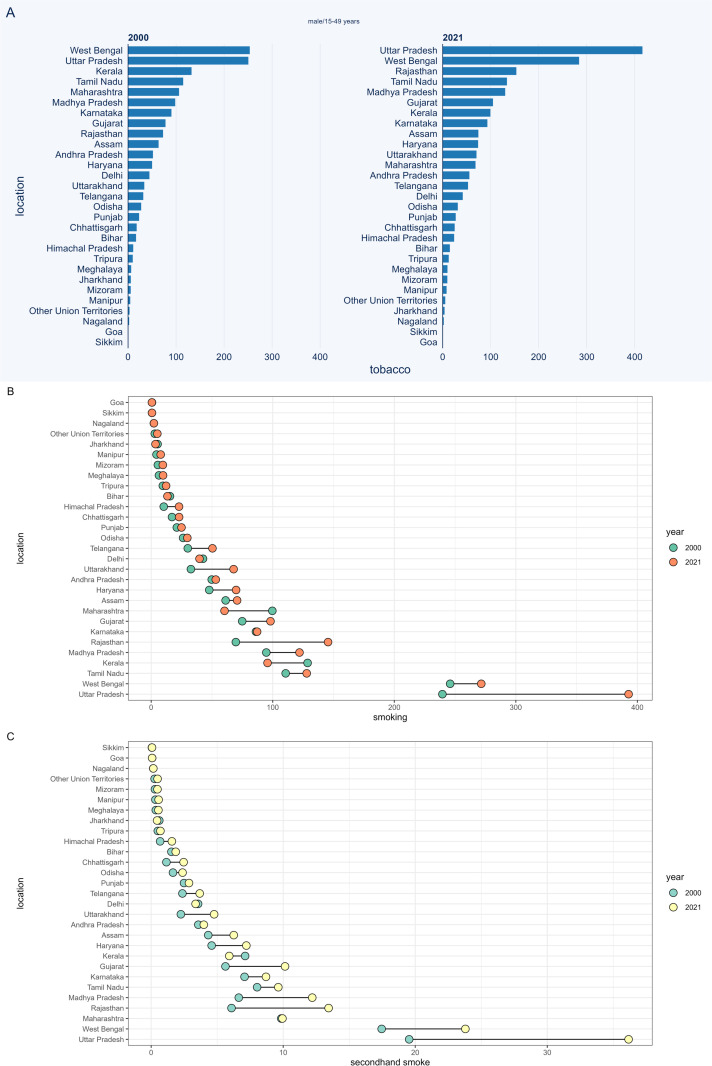
The number of tobacco-related TBL cancer deaths among males aged 15-49 years in different regions of India. (a) tobacco, (b) smoking, (c) second-hand smoke.

**Fig 4:**
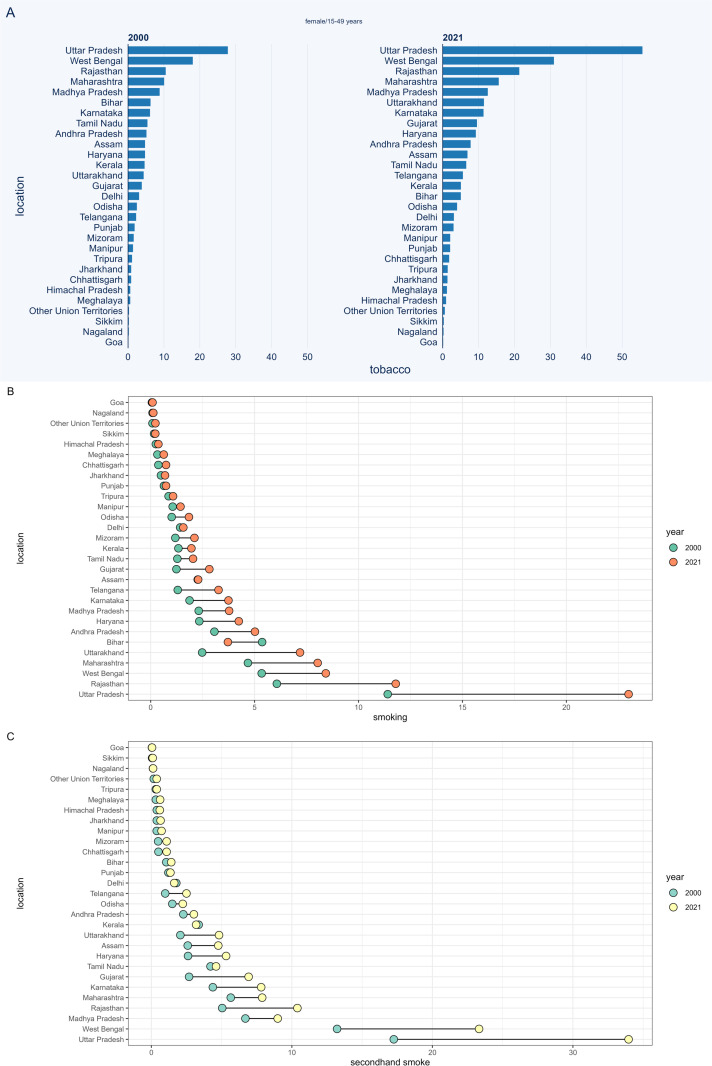
The number of tobacco-related TBL cancer deaths among female aged 15-49 years in different regions of India. (a) tobacco, (b) smoking, (c) second-hand smoke.

Fig 3 displays the difference in TBL cancer deaths among 15–49-year-old males between 2000 and 2021. Uttar Pradesh and West Bengal consistently exhibited the highest burden of tobacco- and secondhand smoke-related TBL cancer deaths in both years. In 2021, Rajasthan surpassed Kerala to rank as the third-highest state in terms of tobacco-related TBL cancer deaths. Rajasthan overtook Maharashtra to become the third-highest state for secondhand smoke-related TBL cancer deaths. Compared to 2000, 5 regions—Delhi, Bihar, Jharkhand, Kerala, and Maharashtra—demonstrated a significant reduction in the burden of deaths. The patterns observed were largely consistent for smoking-related TBL cancer deaths. Additionally, the patterns of change in TBL cancer deaths among women aged 15–49 were similar to those observed in men (Fig 4). The burden of risk factor-related TBL cancer DALYs in the 15–49 age group, as well as deaths and DALYs in the 50–69 and 70 + age groups, are presented in S7-16 Figs.

### APC model analysis of TBL cancer burden due to tobacco in India, 2021

**Fig 5:**
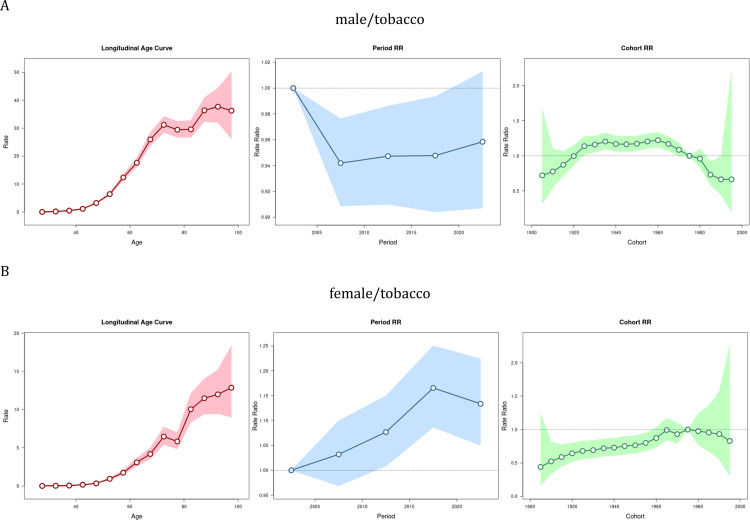
Age-period-cohort analysis of TBL cancer deaths burden associated with tobacco. (A)male, (B)female.

Tobacco-associated TBL cancer mortality risk rose with advancing age, particularly among women. The highest age effect was observed in men aged 90–94 years (Fig 5A), whereas the highest age effect was observed in women aged 95 years or older (Fig 5B). The risk of TBL cancer mortality exhibited fluctuations in the 75–84 age group. The period effect analysis revealed a decline in tobacco-related TBL cancer mortality risk among men after 1994, although the risk remained relatively higher during the 2020–2024 period compared to other periods. The mortality risk among women increased after 2004, peaking between 2015 and 2019. Among men, the cohort effect was highest during the 1920–1975 period, while the lowest effect was observed between 1985 and 1995. Among women, the mortality risk remained relatively low (RR < 1) from 1905 to 1995.

The age-period-cohort effect on the mortality risk of TBL cancer from smoking and secondhand smoke is essentially the same as for tobacco (S17-18 Figs). Women demonstrated a relatively higher risk of smoking-related TBL cancer mortality after 1960. While an increase in secondhand smoke-associated TBL cancer mortality risk was noted after 2014, this trend lacked statistical significance.

### Decomposition analysis of TBL cancer burden attributable to tobacco in India, 2021

**Fig 6:**
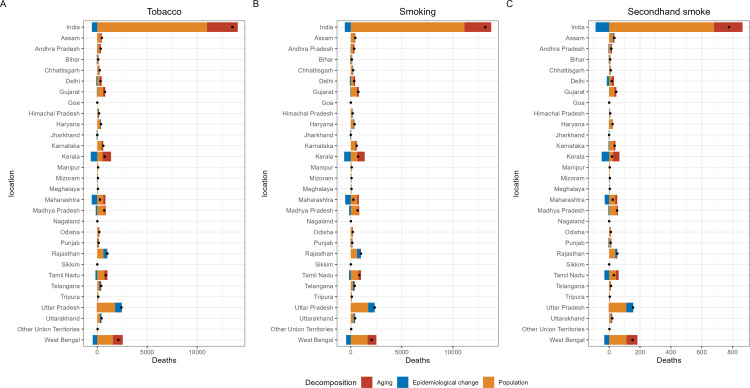
Decomposition analysis of TBL cancer deaths from tobacco exposure in male by region in 2021.

**Fig 7:**
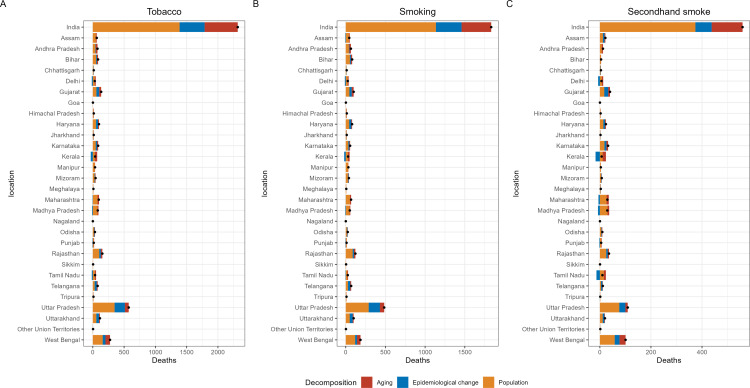
Decomposition analysis of TBL cancer deaths from tobacco exposure in female by region in 2021.

The deaths and DALYs related to tobacco-associated TBL cancer were analyzed through decomposition analysis, as illustrated in Figs 6 and 7 and S19-20 Figs, with gender-specific stratification. Aging and population growth contributed positively to the increase in the burden of tobacco-related TBL cancer deaths in India, while epidemiological changes had a negative effect among males. Epidemiological changes have slowed the increase in tobacco/smoking-related TBL cancer deaths, primarily in the states of West Bengal, Tamil Nadu, Sikkim, Punjab, Odisha, Madhya Pradesh, Maharashtra, Kerala, Jharkhand, Goa, Gujarat, Delhi, Bihar, and Andhra Pradesh. In contrast, the decelerating impact of population aging on tobacco/smoking-related TBL cancer deaths growth was observed only in Uttar Pradesh. In the remaining regions, the increase in TBL cancer deaths was driven by all three factors. It is noteworthy that population aging is the only factor mitigating the increase in tobacco-related TBL cancer death in Uttar Pradesh (Fig 6). Compared to tobacco/smoking related TBL cancer deaths, epidemiological changes also contribute to slowing the growth of tobacco/smoking-related TBL cancer DALYs in Nagaland, Karnataka, and Assam. Similarly, Epidemiological changes were also observed to be a factor mitigating the growth of secondhand smoke-related TBL cancer DALYs burden in Nagaland and Haryana, although this trend was not observed in Odisha (S19 Fig).

The burden of TBL cancer deaths attributable to risk factors among women is presented in Fig 7. Epidemiological changes have contributed to the reduction of tobacco-related TBL cancer deaths in Tripura, Tamil Nadu, Punjab, Nagaland, Madhya Pradesh, Maharashtra, Kerala, Goa, and Delhi. Similarly, epidemiological changes have mitigated the rise in smoking-related TBL cancer deaths in Manipur and Assam. Furthermore, epidemiological changes have mitigated the rise in secondhand smoke-related TBL cancer deaths in Sikkim and Andhra Pradesh, although this trend was not observed in Goa. The DALYs burden of TBL cancer attributable to risk factors among women is shown in S20 Fig. Epidemiological changes have also played a role in alleviating the burden of tobacco-related TBL cancer in Tripura, Tamil Nadu, Sikkim, Punjab, Nagaland, Madhya Pradesh, Maharashtra, Manipur, Kerala, Goa, Delhi, and Assam. Meanwhile, epidemiological changes have effectively curbed the rise in secondhand smoke-associated TBL cancer DALYs in Bihar and Andhra Pradesh.

### Temporal trends in tracheal, bronchus, and lung cancer burden attributable to tobacco

**Fig 8:**
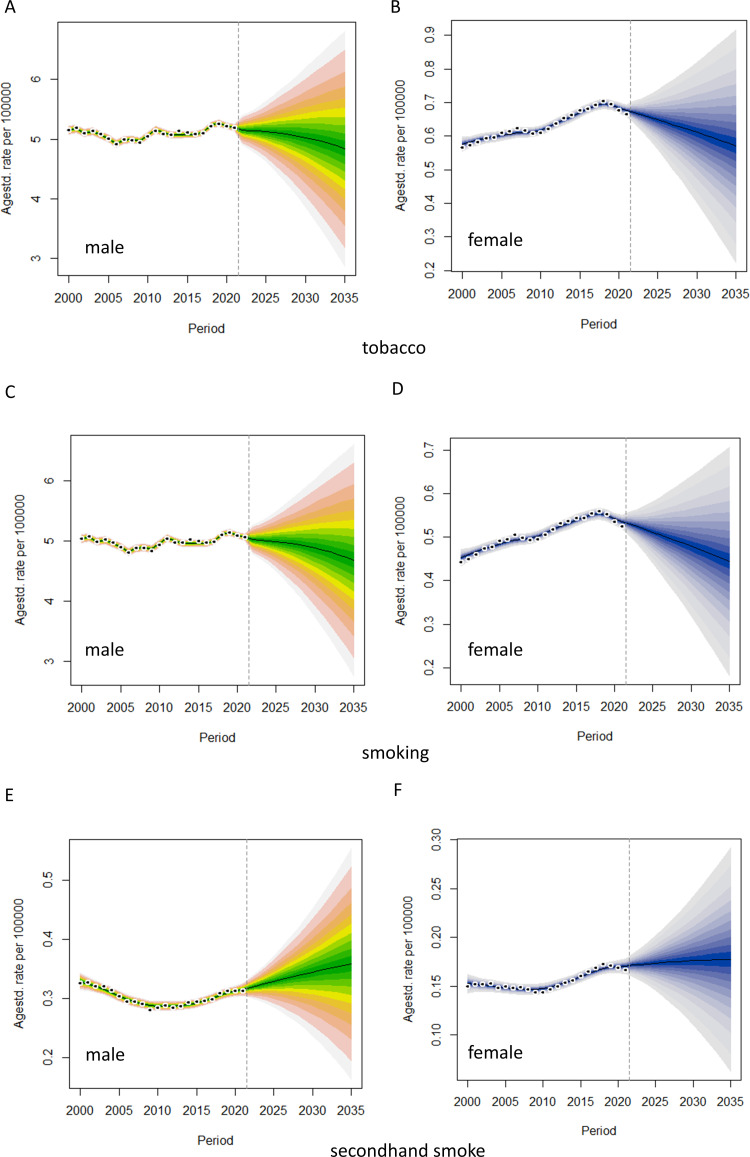
Prediction of age-standardized death rates of TBL cancer from tobacco exposure in India. The rainbow represents male and blue represents female.

By 2035, the age-standardized mortality rates for tobacco- and smoking-related TBL cancer are projected to decline in both males and females (Figs 8A-8D). In contrast, the age-standardized mortality rates for TBL cancer attributable to secondhand smoke is expected to increase among males, while remaining relatively stable among females (Fig 8E). Notably, only the age-standardized DALYs rate for secondhand smoke-related TBL cancer among males demonstrates an upward trend (S21E Fig).

## Discussion

The contribution of all risk factors to mortality increased with age and exhibited a cumulative effect, a finding confirmed by our study. The older the person, the more pathogenic factors that will be caused by the accumulation of unhealthy lifestyles, and the body’s immunity will decline with age. Consequently, elderly individuals are more likely to develop diseases. Currently, India has not only overtaken China as the world’s most populous country but is also facing increasing pressure from rapid population aging [32,33]. Our study further confirms that population growth and aging contribute to the rising burden of tobacco-related TBL cancer in most regions of India.

Since its discovery, tobacco consumption has gone through a long epidemic phase, from its initial popularity in developed countries to market saturation in these areas, and later expanding into developing countries. Tobacco use in India is a complex issue influenced by social, cultural, economic, and policy-related factors. Due to cultural influences, the acceptance of tobacco use is higher in the eastern and northern regions of India [34,35]. The National Family Health Survey (NFHS) revealed that 70% of the population in the northeastern region smokes, which is 26% higher than the national average. Mizoram has the highest tobacco consumption in the region. Additionally, the 2017 Global Adult Tobacco Survey (GATS) indicated an increase in tobacco use in Assam, Tripura, and Manipur [36]. The northeastern states have the highest consumption of betel nuts, as their use is deeply embedded in local customs, tribal culture, and traditions, and is even seen as a sign of respect. The continuous increase in tobacco consumption in the northeast region can be primarily explained by the strong social and cultural acceptance of tobacco use, poorly enforced national policies, greater accessibility of tobacco products, and low literacy levels leading to insufficient understanding of smoking-related risks [37,38]. In contrast, Kerala, with its higher literacy rates, advanced public infrastructure, and leading health development indices, has implemented extensive and socially accepted tobacco control campaigns through the joint efforts of government departments and other stakeholders, significantly reducing tobacco use. In states like Uttar Pradesh, Manipur, and Nagaland, lower female literacy rates correlate with higher smoking rates among women, while states like Kerala, Goa, Maharashtra, and Tamil Nadu, with higher female literacy rates, exhibit lower female smoking rates. The high prevalence of tobacco use in West Bengal is attributed to factors such as public acceptance of chewing tobacco and spitting, the influence of communist politics, intellectuals, and elites who support tobacco use, inadequate enforcement of tobacco control measures by health systems and government departments, and strong resistance from the tobacco industry. Socioeconomic disparities between states are a significant factor in tobacco use rates, with economically disadvantaged groups more likely to use tobacco. Rural populations and those with lower socioeconomic status, who prefer smokeless tobacco (SLT), are the most affected, with tobacco-related illnesses exacerbating their poverty [39,40]. People with less education and lower socioeconomic status are more likely to use tobacco [37]. Exposure to tobacco in schools is a significant factor in the early initiation of smokeless tobacco use among adolescents, and the tobacco use behavior of school teachers also influences adolescents’ tobacco consumption. The Uttarakhand Tobacco Free Initiative (UTFI) aims to protect the state’s youth from tobacco products by implementing education, communication, and training programs in all public schools, government colleges, and women’s colleges to raise awareness about the harms of tobacco. Although the UTFI is a positive initiative, studies have found that while respondents are aware of tobacco bans in schools, enforcement at the implementation level has been ineffective. Additionally, 78% of teachers in Bihar and 31% in Maharashtra are current tobacco users. In Maharashtra, school teachers frequently incorporate tobacco prevention education into their curriculum, whereas in Bihar, such educational initiatives are scarce, and tobacco control policies are nearly absent [41]. Implementing smoking-related education programs in schools can reduce smoking rates among students, and integrating tobacco education into school curricula is a viable approach [42]. The supervisory role of school leaders and teachers, as well as their own non-smoking behavior, is equally crucial. The government and law enforcement agencies must strictly enforce the Tobacco-Free Educational Institution (ToFEI) guidelines and regulate tobacco sales near schools. Furthermore, since most adolescents obtain tobacco from individuals under 21, India could consider raising the legal tobacco purchase age to 21. Notably, the tobacco industry remains a major obstacle to the effective implementation of tobacco control measures. In 2020, India introduced a code of conduct prohibiting officials from collaborating with the tobacco industry, with 22 states, including Karnataka, taking the lead and setting positive examples. Other states should follow suit by adopting similar measures. India’s tobacco tax structure is complex, with low taxation on bidis. The country needs to adopt the World Health Organization’s recommendations to achieve uniform tobacco taxation as soon as possible. Additionally, higher taxes and increased tobacco prices can not only reduce tobacco consumption but also generate funds for tobacco control efforts. Rajasthan imposes the highest taxes on all tobacco products and has implemented innovative campaigns to combat tobacco consumption in schools, police stations, and government offices. In recognition of its efforts, Rajasthan was awarded the WHO Tobacco Control Award in 2019 [43].

With the continuous development of the tobacco industry, e-cigarettes began to appear in the public eye in 2003 [44]. In 2016–2017, the e-cigarette usage rate in India was 0.1%, with a crude prevalence rate of 0.02% [45]. Government tobacco control measures, including the Prevention of Electronic Crimes (PECA) Act, prohibit all forms of e-cigarettes as well as any media promotion or sale of e-cigarettes. Despite penalties for violating the policy, e-cigarettes can still be seen being sold through retail stores, underground markets, and online platforms in major Indian cities such as Bangalore, Chandigarh, and Delhi [44]. This may be due to limited public understanding of the policy in India and insufficient enforcement and supervision across regions. The study show that only 30% of Indians are aware of the PECA Act. Punjab banned e-cigarettes in 2013, and states like Haryana, Kerala, Mizoram, Karnataka, Bihar, and Jammu and Kashmir have also prohibited e-cigarettes [46]. It is noteworthy that internet platforms provide a favorable environment for the marketing and sale of e-cigarettes. Despite multiple bans on e-cigarettes in India, 26% of Indian youth are still exposed to e-cigarette marketing through online platforms. Tobacco marketers are also promoting e-cigarettes by leveraging influencers [44]. In early 2023, the Indian government announced new guidelines targeting influencers to prevent potential e-cigarette promotions, thereby protecting consumers from misleading marketing advertisements. Non-compliance with these guidelines could result in fines of up to 500,000 rupees [47]. Currently, the Tobacco Enforcement and Reporting Campaign (TERM) has successfully established a system to monitor online tobacco marketing, which is now operational in India to counteract the industry’s digital promotional efforts. Smokeless tobacco is the most prevalent form of tobacco use in India, with commonly used products including khaini, gutkha, and betel nut. Smokeless tobacco in India is used by one-fifth of the population aged 15 and above due to its easy availability, low cost, and attractive packaging. It is often perceived as less harmful than smoking and serves as a short-term substitute for smoking in smoke-free environments. A representative national survey indicates that the prevalence of smokeless tobacco in India increased from 15% in 1987 to 23.4% in 2005 [48]. In 2012, the central government of India imposed a ban on Gutkha products and has continuously strengthened enforcement in more states, leading to a sustained decline in the market for smokeless tobacco products. As the years of formal education increase for both women and men, the prevalence of SLT use gradually decreases. The use of SLT is culturally and socially accepted across all ages and genders, which is distinctly different from smoking tobacco products. This is one of the reasons for the widespread use of SLT among Indian women, with illiterate women using smokeless tobacco more frequently [49,50]. The tobacco control policies implemented by the Indian government, such as the ban on the sale of gutkha and smoke-free institution guidelines, have shown significant effectiveness. The prevalence of smokeless tobacco in India decreased from 25.9% in GATS-1 to 21.4% in GATS-2, and the proportion of adolescents aged 13–15 using SLT declined from 14% in 2003 to 4.1% in 2019 [43]. The tobacco industry claims that Heated Tobacco Products (HTP) are a safer alternative to traditional tobacco, attracting some teenagers, smokers, and non-smokers who prefer real cigarettes but are unwilling to try e-cigarettes. The stylish design and diverse flavors of HTP are marketing strategies aimed at capturing the attention of young people. Moreover, users of HTP believe that it is more socially acceptable, less harmful, and less addictive [51,52]. Through comprehensive marketing strategies, the tobacco industry has successfully captured a share of the market for HTP. Since the product was improved in 2015, its popularity among consumers has continued to rise. There is limited research on the current prevalence of HTP in India, but based on trends in other countries, India is likely to become a key target for the tobacco industry. If India strengthens regulations on e-cigarettes, HTP could emerge as an alternative, which is something the Indian government needs to be vigilant about.

Since the 1930s, awareness of the association between lung cancer and tobacco use has grown steadily, and by the 1950s, tobacco exposure had been globally recognized as a significant risk factor for lung cancer [33]. However, it was not until 1964 that the U.S. Department of Health published the first official report on smoking and health, confirming the link between smoking and lung cancer. In 2000, India banned tobacco advertising in state-controlled electronic media and publications. In 2003, India signed the Framework Convention on Tobacco Control. In the same year, India enacted the Cigarettes and Other Tobacco Products Act 2003, which was implemented in 2004 [53]. To further strengthen the tobacco control effects of these acts, India initiated a pilot National Tobacco Control Program (NTCP) in 2007–2008, which operates at the national, state, and district levels and formulates specific tobacco control measures at different levels [54]. In addition, India’s smoking cessation initiative aims to persuade people to quit smoking by sending text messages via mobile phones [8]. The GATS survey discovered that since the implementation of NTCP, during the period of 2009–2010 and 2016–2017, the tobacco epidemic rate in India dropped by 28.6%, from 34.6%, and nearly 8.1 million tobacco users decreased [8,54]. The low penalty amounts stipulated under COTPA represent another significant barrier to policy enforcement. Increasing fines to deter tobacco sales and consumption is an urgent priority. Karnataka even enacted state laws against smoking before the national COTPA was introduced and is one of the few states demonstrating political will by actively implementing COTPA measures [55]. India’s Cigarette Act of 1975 mandates that the warning “Smoking is harmful to health” be displayed on all cigarette packaging, paper cigarette containers, and cigarette advertisements [53,56]. In 2015, India implemented a regulation requiring graphic health warnings to cover 85% of all tobacco product packaging. Research indicates that tobacco users comprehend and endorse the graphic health warnings on tobacco packaging. Nonetheless, merely 20% of non-tobacco users stated that such warnings discouraged them from cigarette use [57]. Effective December 1, 2022, the Indian government required that the text on tobacco products be updated to the more alarming warning: “TOBACCO USERS DIE YOUNGER” [58]. Additionally, implementing plain packaging, as demonstrated in other countries, may help decrease tobacco use in India.

Studies have demonstrated that at different levels of smoking exposure, women consistently have a higher risk of developing major types of lung cancer than men, possibly due to women’s greater sensitivity to tobacco carcinogens [59–62]. Factors such as genetic predisposition, exposure to sex hormones, and molecular characteristics may further increase women’s susceptibility to lung cancer [2,63]. Economic globalization and related social changes have gradually integrated women into the labor market, making them a significant part of the workforce. For instance, Gujarat, the second-largest tobacco producer in India, has a high rate of female employment in the tobacco industry [37]. Additionally, the tobacco industry has strategically targeted women as potential consumers. Throughout the 20th century, the industry employed various marketing tactics, from promoting tobacco as a weight-loss aid to framing smoking as a symbol of gender equality, to encourage women to smoke [39,64]. This may explain the cohort effect observed in our study, which indicates a progressively increased risk of lung cancer mortality among women born before 1965 due to tobacco exposure. Furthermore, the tobacco industry is increasingly focusing on women in low- and middle-income countries. The study conducted by Goel et al. documented a significant increase in smoking prevalence among Indian women, rising from 1.4% in 2005 to 2.9% by 2010. This upward trend continued, with data from 2012 indicating a further increase to 3.2%. However, the GATS-2 reported an overall tobacco use rate of 9% among women, while the smoking rate showed a decline compared to previous period, specifically standing at 2% [54,65]. This trend is corroborated by the GATS and NHFS-5, which reported a growing cycle effect of tobacco use among Indian women [54]. These factors may account for the period effect observed in our study, which shows an increased risk of lung cancer mortality attributable to tobacco use among women after 2004. In the cultural context of India, it is socially unacceptable for women to smoke. Smoking among women is frequently met with disapproval from society and carries substantial social stigma. As a result, many female smokers conceal their smoking habits from family members, parents, and even healthcare providers [66–68]. A study conducted in India observed that women experienced feelings of guilt associated with being SLT users and exhibited hesitation or even avoidance when questioned about their SLT usage [69]. Additionally, some women reported experiencing guilt related to smoking due to their roles as mothers [70]. In South Korea, women have been known to smoke in private but publicly deny smoking [71]. Consequently, due to sociocultural and personal factors, it is possible that smoking among Indian women may be underreported, which could lead to an underestimation of female smoking rates.

Exposure to secondhand smoke has been recognized as a significant health risk factor. In our study, the burden of lung cancer caused by secondhand smoke was relatively small compared to that caused by direct smoking; however, the prediction model indicated that the burden of lung cancer attributable to secondhand smoke is projected to increase in the future. Non-smoking individuals exposed to secondhand smoke face a 16% higher risk of developing cancer [6,7]. In India, the prevalence of secondhand smoke exposure stands at 29.5%. Furthermore, a national survey on tobacco use in India revealed that 11.2% of households include smokers [72]. A meta-analysis demonstrated that children with smoking parents are up to 13 times more likely to be exposed to secondhand smoke [73]. Additionally, parental education levels were found to be inversely associated with the risk of secondhand smoke exposure [74]. It was estimated that approximately 23% of adults in India are exposed to secondhand smoke in public places [75]. Government buildings, healthcare facilities, restaurants, and public transportation are the four public spaces with the highest levels of secondhand smoke exposure [76]. Although the COTPA and related legislation enacted in 2008 prohibit smoking in public places, they do not ban smoking in designated areas or “smoking zones” in certain public spaces such as restaurants, hotels, and airports. In fact, when some public places provide designated enclosed smoking rooms under very strict conditions, it does not contribute to achieving 100% smoke-free indoor environments [77–79]. Therefore, it is essential to completely prohibit smoking in all public places. The Indian government has implemented tobacco control policies prohibiting smoking in public places, leading to the installation of no-smoking signage in establishments such as restaurants, bars, hospitals, canteens, and government buildings. Short-term studies have indicated improvements in air quality in bars, restaurants, and pubs following the enforcement of smoke-free laws [80]. Bangalore serves as an exemplary model for its significant contributions to enforcing smoke-free public spaces and improving public adherence to anti-smoking regulations. From 2017 to 2023, the city implemented comprehensive tobacco control measures, including enforcement activities, training programs for law enforcement personnel, the removal of designated smoking zones, the installation of “No Smoking” signs, and public awareness campaigns highlighting the risks of tobacco use and secondhand smoke exposure. These efforts have yielded remarkable outcomes, with a local study reporting a nearly 27% decline in public smoking and a 25% increase in the number of “No Smoking” signs displayed in public areas [81]. As tobacco control policies have been implemented, the 2017 Global Tobacco Epidemic Report noted that approximately 60–70% of public spaces in India were smoke-free, and a comparison of GATS-1 and GATS-2 reports revealed a significant decline in tobacco exposure rates in public places [76]. According to the GATS-2 report, workplaces represent a significant setting for secondhand smoke exposure [77]. Working adults spend a substantial portion of their time in workplaces, making these environments one of the primary sources of SHS exposure for non-smokers. In India, nearly one-third of non-smokers are exposed to SHS, with approximately 30% of adults facing secondhand smoke exposure in their workplaces [75]. A comparison of the GATS-1 and GATS-2 reports shows that the prevalence of secondhand smoke exposure in Indian workplaces has seen negligible change, underscoring the urgent need to strengthen smoking management in these settings [76]. Enterprises should foster a smoke-free culture by incorporating smoke-free policies into their regulations and imposing fines for violations. Additionally, regular secondhand smoke awareness campaigns and support such as cessation courses and psychological counseling can enhance employee health awareness and reduce smoking rates. The smoke-free initiatives in rural areas of Maharashtra demonstrate that government and community collaboration can overcome significant challenges to achieve effective tobacco control. Therefore, tobacco control requires the collective participation of multiple stakeholders from both government and private sectors. The government must also provide more comprehensive cessation services, including increasing the number of cessation clinics/centers and training cessation counselors.

In summary, India’s tobacco control mission is challenging and long-term. In the short term, strengthening legal policies, tax reforms, and enhancing enforcement are essential to curb tobacco consumption growth. In the long term, agricultural transformation and international cooperation are needed to address structural challenges. Meanwhile, the government should continuously monitor progress toward smoke-free goals, regularly evaluate policy effectiveness, and make dynamic adjustments. Finally, the issue of tobacco use among women requires urgent attention, particularly in the northeastern states, and gender-specific strategies should be incorporated into tobacco control planning.

Our study has several limitations. First, our data were entirely sourced from the Global Burden of Disease database, and the availability and quality of these data significantly influence the accuracy of disease burden estimates. Additionally, since the database does not provide further classification of lung cancer, we were unable to analyze specific lung cancer subtypes.

## Conclusion

Despite the decline in the global tobacco epidemic, the burden of TBL cancer attributable to tobacco remains high in certain regions of India. Regional governments must develop and implement locally appropriate measures to reduce the burden of tobacco-related TBL cancer.

## Supporting information

S1 Table The burden of TBL cancer deaths associated with tobacco exposure among male in India in 2000 and 2021 and the temporal trend from 2000 to 2021. (DOCX)

S2 Table The burden of TBL cancer deaths associated with tobacco exposure among female in India in 2000 and 2021 and the temporal trend from 2000 to 2021. (DOCX)

S3 Table The burden of TBL cancer DALYs associated with tobacco exposure among male in India in 2000 and 2021 and the temporal trend from 2000 to 2021. (DOCX)

S4 Table The burden of TBL cancer DALYs associated with tobacco exposure among female in India in 2000 and 2021 and the temporal trend from 2000 to 2021. (DOCX)

S1 Fig Tobacco exposure related lung cancer death rates among male by age in different regions in 2021. (TIF)

S2 Fig Tobacco exposure related lung cancer death rates among female by age in different regions of in 2021. (TIF)

S3 Fig Tobacco exposure related lung cancer DALYs among male by age in different regions of India in 2021. (TIF)

S4 Fig Tobacco exposure related lung cancer DALYs rates among male by age in different regions of India in 2021. (TIF)

S5 Fig Tobacco exposure related lung cancer DALYs among female by age in different regions of India in 2021. (TIF)

S6 Fig Tobacco exposure related lung cancer DALYs rates among female by age in different regions of India in 2021. (TIF)

S7 Fig The number of tobacco-related TBL cancer deaths among males aged 50–69 years in different regions. (a) tobacco, (b) smoking, (c) second-hand smoke.
(TIF)

S8 Fig The number of tobacco-related TBL cancer deaths among females aged 50–69 years in different regions. (a) tobacco, (b) smoking, (c) second-hand smoke.
(TIF)

S9 Fig The number of tobacco-related TBL cancer deaths among males aged 70 + years in different regions. (a) tobacco, (b) smoking, (c) second-hand smoke.
(TIF)

S10 Fig The number of tobacco-related TBL cancer deaths among females aged 70 + years in different regions. (a) tobacco, (b) smoking, (c) second-hand smoke.
(TIF)

S11 Fig The number of tobacco-related TBL cancer DALYs among males aged 15–49 years in different regions. (a) tobacco, (b) smoking, (c) second-hand smoke.
(TIF)

S12 Fig The number of tobacco-related TBL cancer DALYs among females aged 15–49 years in different regions. (a) tobacco, (b) smoking, (c) second-hand smoke.
(TIF)

S13 Fig The number of tobacco-related TBL cancer DALYs among males aged 50–69 years in different regions. (a) tobacco, (b) smoking, (c) second-hand smoke.
(TIF)

S14 Fig The number of tobacco-related TBL cancer DALYs among females aged 50–69 years in different regions. (a) tobacco, (b) smoking, (c) second-hand smoke.
(TIF)

S15 Fig The number of tobacco-related TBL cancer DALYs among males aged 70 + years in different regions. (a) tobacco, (b) smoking, (c) second-hand smoke.
(TIF)

S16 Fig The number of tobacco-related TBL cancer DALYs among females aged 70 + years in different regions. (a) tobacco, (b) smoking, (c) second-hand smoke.
(TIF)

S17 Fig Age-period-cohort analysis of TBL cancer deaths burden associated with smoking. (A)male, (B)female.
(TIF)

S18 Fig Age-period-cohort analysis of TBL cancer deaths burden associated with secondhand smoke. (A)male, (B)female.
(TIF)

S19 Fig Decomposition analysis of TBL cancer DALYs from tobacco exposure in male in 2021. (TIF)

S20 Fig Decomposition analysis of TBL cancer DALYs from tobacco exposure in female in 2021. (TIF)

S21 Fig Prediction of age-standardized DALYs rates of TBL cancer from tobacco exposure in India. the rainbow represents male and blue represents female. (TIF)

## References

[1] Tobacco [Internet]. [cited 2025 Mar 6]. Available from: https://www.who.int/news-room/fact-sheets/detail/tobacco

[2] Deng Y, Zhao P, Zhou L, Xiang D, Hu J, Liu Y, et al. Epidemiological trends of tracheal, bronchus, and lung cancer at the global, regional, and national levels: a population-based study. J Hematol Oncol. 2020 Jul 20;13(1):98. 32690044 10.1186/s13045-020-00915-0 PMC7370495

[3] Sheng L, Tu J-W, Tian J-H, Chen H-J, Pan C-L, Zhou R-Z. A meta-analysis of the relationship between environmental tobacco smoke and lung cancer risk of nonsmoker in China. Medicine (Baltimore). 2018;97(28):e11389. doi: 10.1097/MD.0000000000011389 29995781 PMC6076103

[4] Wu X, Zhu B, Xu S, Liu Y, Bi Y, Zhou B. A comparison of the burden of lung cancer attributable to tobacco exposure in China and the USA. Ann Transl Med. 2020;8(21):1412. doi: 10.21037/atm-20-996 33313157 PMC7723584

[5] Tobacco Smoking and Mortality in Asia: A Pooled Meta-analysis - PubMed [Internet]. [cited 2025 Mar 6]. Available from: https://pubmed.ncbi.nlm.nih.gov/30924901/

[6] Salerno PRV de O, Palma Dallan LA, Rodrigues Pereira GT, Pego Fernandes PM, Mingarini Terra R, Rajagopalan S, et al. Trends in tracheal, bronchial and lung cancer attributed to smoking in South America: Global Burden of Disease analysis 1990-2019. Rev Panam Salud Publica. 2024;48:e30. doi: 10.26633/RPSP.2024.30 38576842 PMC10993800

[7] Exposure to Secondhand Smoke and Risk of Cancer in Never Smokers: A Meta-Analysis of Epidemiologic Studies-PubMed [Internet]. [cited 2025 Mar 6]. Available from: https://pubmed.ncbi.nlm.nih.gov/30208628/10.3390/ijerph15091981 PMC6164459 30208628

[8] Kulothungan V, Ramamoorthy T, Sarveswaran G, Jadhav SY, Mathur P. Association of Tobacco Use and Cancer Incidence in India: A Systematic Review and Meta-Analysis. JCO Glob Oncol. 2024;10:e2400152. doi: 10.1200/GO.24.00152 39173081

[9] Kumari N, Manisha M, Paul S, Ram R. Socioeconomic inequality among smoking and smokeless tobacco uses among males in India: a decomposition analysis. Public Health. 2024;227:176–86. doi: 10.1016/j.puhe.2023.12.006 38232566

[10] India State-Level Disease Burden Initiative Cancer Collaborators. The burden of cancers and their variations across the states of India: the Global Burden of Disease Study 1990-2016. Lancet Oncol. 2018;19(10):1289–306. doi: 10.1016/S1470-2045(18)30447-9 30219626 PMC6167407

[11] Sharma R, Rakshit B. Global burden of cancers attributable to tobacco smoking, 1990-2019: an ecological study. EPMA J. 2022;14(1):167–82. doi: 10.1007/s13167-022-00308-y 36866162 PMC9971393

[12] Zhu Z, Ye W, Zhang L, Jia W, Chen B, Wang Q, et al. Diversities of disability caused by lung cancer in the 66 Belt and Road initiative countries: a secondary analysis from the Global Burden of Disease Study 2019. Front Oncol. 2023;13:1247006. doi: 10.3389/fonc.2023.1247006 38023230 PMC10668146

[13] GBD 2019 Chronic Respiratory Diseases Collaborators. Global burden of chronic respiratory diseases and risk factors, 1990-2019: an update from the Global Burden of Disease Study 2019. EClinicalMedicine. 2023;59:101936. doi: 10.1016/j.eclinm.2023.101936 37229504 PMC7614570

[14] Guo Q, Lu Y, Liu W, Lan G, Lan T. The global, regional, and national disease burden of breast cancer attributable to tobacco from 1990 to 2019: a global burden of disease study. BMC Public Health. 2024;24(1):107. doi: 10.1186/s12889-023-17405-w 38184557 PMC10770986

[15] GBD 2021 Causes of Death Collaborators. Global burden of 288 causes of death and life expectancy decomposition in 204 countries and territories and 811 subnational locations, 1990-2021: a systematic analysis for the Global Burden of Disease Study 2021. Lancet. 2024 May 18;403(10440):2100–32. 38582094 10.1016/S0140-6736(24)00367-2 PMC11126520

[16] GBD 2021 Diseases and Injuries Collaborators. Global incidence, prevalence, years lived with disability (YLDs), disability-adjusted life-years (DALYs), and healthy life expectancy (HALE) for 371 diseases and injuries in 204 countries and territories and 811 subnational locations, 1990-2021: a systematic analysis for the Global Burden of Disease Study 2021. Lancet. 2024 May 18;403(10440):2133–61. 38642570 10.1016/S0140-6736(24)00757-8 PMC11122111

[17] Global burden and strength of evidence for 88 risk factors in 204 countries and 811 subnational locations, 1990-2021: a systematic analysis for the Global Burden of Disease Study 2021-PubMed [Internet]. [cited 2025 Mar 7]. Available from: https://pubmed.ncbi.nlm.nih.gov/38762324/10.1016/S0140-6736(24)00933-4 PMC11120204 38762324

[18] Ilic M, Ilic I. Cancer mortality in Serbia, 1991-2015: an age-period-cohort and joinpoint regression analysis. Cancer Commun (Lond). 2018;38(1):10. doi: 10.1186/s40880-018-0282-3 29764495 PMC5993142

[19] Bai Z, Wang H, Shen C, An J, Yang Z, Mo X. The global, regional, and national patterns of change in the burden of non-malignant upper gastrointestinal diseases from 1990 to 2019 and the forecast for the next decade. Int J Surg. 2024 Jul 3;111(1):80–92. 10.1097/JS9.0000000000001902 PMC11745775 38959095

[20] Analysis of the Global Burden of Disease study highlights the global, regional, and national trends of chronic kidney disease epidemiology from 1990 to 2016 - PubMed [Internet]. [cited 2025 Jan 17]. Available from: https://pubmed.ncbi.nlm.nih.gov/30078514/

[21] Das Gupta P. Standardization and decomposition of rates from cross-classified data. Genus. 1994;50(3–4):171–96. 12319256

[22] Chevan A, Sutherland M. Revisiting Das Gupta: refinement and extension of standardization and decomposition. Demography. 2009;46(3):429–49. doi: 10.1353/dem.0.0060 19771938 PMC2831344

[23] Global temporal trends and projections of acute hepatitis E incidence among women of childbearing age: Age-period-cohort analysis 2021 - Journal of Infection [Internet]. [cited 2025 Mar 7]. Available from: https://www.journalofinfection.com/article/S0163-4453(24)00184-1/fulltext 10.1016/j.jinf.2024.106250 39181413

[24] Li H, Yang X, Zhang A, Liang G, Sun Y, Zhang J. Age-period-cohort analysis of incidence, mortality and disability-adjusted life years of esophageal cancer in global, regional and national regions from 1990 to 2019. BMC Public Health. 2024 Jan 17;24(1):212. 38233775 10.1186/s12889-024-17706-8 PMC10795420

[25] Zou Z, Liu G, Hay SI, Basu S, Belgaumi UI, Dhali A, et al. Time trends in tuberculosis mortality across the BRICS: an age-period-cohort analysis for the GBD 2019. eClinicalMedicine. 2022 Sep 17;53:101646. 10.1016/j.eclinm.2022.101646 PMC9486016 36147625

[26] Xie Z, Chen S, He C, Cao Y, Du Y, Yi L, et al. Trends and age-period-cohort effect on the incidence of falls from 1990 to 2019 in BRICS. Heliyon. 2024;10(5):e26771. doi: 10.1016/j.heliyon.2024.e26771 38434415 PMC10907765

[27] Global, regional, and national thyroid cancer age-period-cohort modeling and Bayesian predictive modeling studies: A systematic analysis of the global burden of disease study 2019-PubMed [Internet]. [cited 2025 Mar 7]. Available from: https://pubmed.ncbi.nlm.nih.gov/38045179/10.1016/j.heliyon.2023.e22490 PMC10689957 38045179

[28] Zeng Q, Jiang D. Global trends of interstitial lung diseases from 1990 to 2019: an age-period-cohort study based on the Global Burden of Disease study 2019, and projections until 2030. Front Med (Lausanne). 2023;10:1141372. doi: 10.3389/fmed.2023.1141372 37554509 PMC10404716

[29] Sun S, Zhang T, Yu H, Xia T, Yao Y, Sun M, et al. Time trends in Alzheimer’s disease mortality attributable to metabolic risks and smoking in China from 1990 to 2019: an age-period-cohort analysis. Front Aging Neurosci. 2024;16:1425577. doi: 10.3389/fnagi.2024.1425577 39026988 PMC11256009

[30] Ma J, Song Y, Bai X-M. Global, regional, and national burden and trends of early-onset tracheal, bronchus, and lung cancer from 1990 to 2019. Thorac Cancer. 2024;15(8):601–13. doi: 10.1111/1759-7714.15227 38303633 PMC10928250

[31] Wang S, Dong Z, Wan X. Global, regional, and national burden of inflammatory bowel disease and its associated anemia, 1990 to 2019 and predictions to 2050: An analysis of the global burden of disease study 2019. Autoimmun Rev. 2024;23(3):103498. doi: 10.1016/j.autrev.2023.103498 38052263

[32] India Overtakes China as the World’s Most Populous Country | United Nations iLibrary [Internet]. [cited 2025 Mar 7]. Available from: https://www.un-ilibrary.org/content/papers/10.18356/27081990-153

[33] The global burden of lung cancer: current status and future trends - PubMed [Internet]. [cited 2025 Mar 7]. Available from: https://pubmed.ncbi.nlm.nih.gov/37479810/

[34] Ghate N, Kumar P, Dhillon P. Socioeconomic determinants of smokeless tobacco use among Indian women: An analysis of global adult tobacco survey-2, India. WHO South East Asia J Public Health. 2022;11(1):24–31. doi: 10.4103/WHO-SEAJPH.WHO-SEAJPH_160_21 36308270

[35] Verma M, Rana K, Bhatt G, Sharma N, Lal P. Trends and determinants of tobacco use initiation in India: analysis of two rounds of the Global Adult Tobacco Survey. BMJ Open. 2023;13(9):e074389. doi: 10.1136/bmjopen-2023-074389 37739473 PMC10533663

[36] Ngaihte P, Zomawia E, Kaushik I. Cancer in the NorthEast India: Where we are and what needs to be done?. Indian J Public Health. 2019;63(3):251–3. doi: 10.4103/ijph.IJPH_323_18 31552857

[37] Shaikh R, Janssen F, Vogt T. The progression of the tobacco epidemic in India on the national and regional level, 1998-2016. BMC Public Health. 2022 Feb 15;22(1):317. 35168590 10.1186/s12889-021-12261-y PMC8845293

[38] Malhotra RK, Manoharan N, Nair O, Deo SVS, Rath GK. Trends and future burden of tobacco-related cancers incidence in Delhi urban areas: 1988-2012. Indian J Public Health. 2019;63(1):33–8. doi: 10.4103/ijph.IJPH_91_18 30880735

[39] Goel S, Tripathy JP, Singh RJ, Lal P. Smoking trends among women in India: Analysis of nationally representative surveys (1993-2009). South Asian J Cancer. 2014 Oct;3(4):200–2. 25422803 10.4103/2278-330X.142958 PMC4236695

[40] Jayakrishnan R, Geetha S, Binukumar B, , Lekshmi K. Self-reported tobacco use, knowledge on tobacco legislation and tobacco hazards among adolescents in rural Kerala State. Indian J Dent Res. 2011;22(2):195–9. doi: 10.4103/0970-9290.84280 21891884

[41] Garcia de Quevedo I, Arrazola RA, Yadav R, Soura BD, Ahluwalia IB. Implementation of the Uttarakhand Tobacco Free Initiative in Schools, India, 2016. Prev Chronic Dis. 2021 Jul 29;18:E74. 34324415 10.5888/pcd18.200650 PMC8388200

[42] Pahari S, Sivanantham P, Kar SS. Adherence to the National Tobacco-Free School Policy in Selected Schools of Puducherry District in India: A Cross-Sectional Exploratory Study. Cureus. 2024;16(2):e53984. doi: 10.7759/cureus.53984 38476790 PMC10927484

[43] Lahoti S, Dixit P. Declining trend of smoking and smokeless tobacco in India: A decomposition analysis. PLoS One. 2021;16(2):e0247226. doi: 10.1371/journal.pone.0247226 33630963 PMC7906458

[44] Bahl D, Bassi S, Thapliyal N, Aneja K, Sinha P, Arora M. Unveiling the Digital Landscape of E-Cigarette Marketing in India: Evidence From Mixed Method Study. Tob Use Insights. 2024;17:1179173X241264504. doi: 10.1177/1179173X241264504 39552925 PMC11568514

[45] Amalia B, Kapoor S, Sharma R, Singh RJ. E-cigarette retailer storefront availability following a nationwide prohibition of e-cigarettes in India: A multicentric compliance assessment. Tob Prev Cessat. 2020;6:42. doi: 10.18332/tpc/123822 33083675 PMC7549508

[46] Relita Mendonca R, Narayanan VA, Sandeep DS, Ruman A, Charyulu RN. Regulating E-cigarettes in India: A conundrum for the global giant in tobacco production. Indian J Tuberc. 2019;66(2):288–93. doi: 10.1016/j.ijtb.2019.02.014 31151498

[47] Phua J, Jin SV, Hahm JM. Celebrity-endorsed e-cigarette brand Instagram advertisements: Effects on young adults’ attitudes towards e-cigarettes and smoking intentions. J Health Psychol. 2018;23(4):550–60. doi: 10.1177/1359105317693912 28810409

[48] Suliankatchi Abdulkader R, Sinha DN, Jeyashree K, Rath R, Gupta PC, Kannan S, et al. Trends in tobacco consumption in India 1987-2016: impact of the World Health Organization Framework Convention on Tobacco Control. Int J Public Health. 2019 Jul;64(6):841–51. 31134319 10.1007/s00038-019-01252-x

[49] Declining trends in smokeless tobacco use among Indian women: findings from global adult tobacco survey I and II - PubMed [Internet]. [cited 2025 Mar 7]. Available from: https://pubmed.ncbi.nlm.nih.gov/34753440/10.1186/s12889-021-12089-6 PMC8576912 34753440

[50] Nair S, Singh L, Deepani V, Alee NT, Sharma S, Ovung S, et al. Predictors of smokeless tobacco use among the adult population of north-east India during 2009-2017: A decomposition analysis. Indian J Med Res. 2022;156(2):330–8. doi: 10.4103/ijmr.ijmr_3229_21 36629193 PMC10057354

[51] Sun T, Anandan A, Lim CCW, East K, Xu SS, Quah ACK, et al. Global prevalence of heated tobacco product use, 2015-22: A systematic review and meta-analysis. Addiction. 2023;118(8):1430–44. doi: 10.1111/add.16199 37005862

[52] Peruga A, Rodríguez Lozano F, López MJ, Córdoba García R, Nerín I, Sureda X, et al. Tobacco heated products: a new challenge in tobacco control. Gac Sanit. 2022;36(1):57–9. doi: 10.1016/j.gaceta.2020.12.033 33563478

[53] Kaur J, Jain DC. Tobacco control policies in India: implementation and challenges. Indian J Public Health. 2011;55(3):220–7. doi: 10.4103/0019-557X.89941 22089690

[54] Pahari S, Barman D, Talukdar R. Tobacco usage in India: A meta-analysis of evidence drawn from regional studies between 2010 and 2022. Trop Med Int Health. 2023;28(9):699–709. doi: 10.1111/tmi.13924 37583260

[55] Hebbar PB, Bhojani U, Kennedy J, Rao V. From Policy to Practice: Lessons from Karnataka about Implementation of Tobacco Control Laws. Indian J Community Med. 2017;42(2):77–80. doi: 10.4103/0970-0218.205212 28553022 PMC5427866

[56] Rao V, Chaturvedi P. Tobacco and health in India. Indian J Cancer. 2010;47(Suppl 1):3–8. doi: 10.4103/0019-509X.64373 20622406

[57] Mudey A, Shukla A, Choudhari SG, Joshi A. Does the Graphic Health Warning on Tobacco Products Have an Influence on Tobacco Consumers in India? A Scoping Review. Cureus. 2023 Apr;15(4):e38304. 37255891 10.7759/cureus.38304 PMC10226759

[58] New Specified Health Warning on Tobacco Products packs [Internet]. [cited 2025 Mar 7]. Available from: https://www.gkseries.com/blog/new-specified-health-warning-on-tobacco-products-packs/

[59] Zang EA, Wynder EL. Differences in lung cancer risk between men and women: examination of the evidence. J Natl Cancer Inst. 1996 Feb 21;88(3–4):183–92. 8632492 10.1093/jnci/88.3-4.183

[60] Harris RE, Zang EA, Anderson JI, Wynder EL. Race and sex differences in lung cancer risk associated with cigarette smoking. Int J Epidemiol. 1993 Aug;22(4):592–9. 8225730 10.1093/ije/22.4.592

[61] The association between smoking quantity and lung cancer in men and women - PubMed [Internet]. [cited 2025 Mar 7]. Available from: https://pubmed.ncbi.nlm.nih.gov/22797799/10.1378/chest.12-1068 22797799

[62] Rezaei F, Mazidimoradi A, Rayatinejad A, Allahqoli L, Salehiniya H. Temporal trends of tracheal, bronchus, and lung cancer between 2010 and 2019, in Asian countries by geographical region and sociodemographic index, comparison with global data. Thorac Cancer. 2023;14(18):1668–706. doi: 10.1111/1759-7714.14912 37127553 PMC10290923

[63] Worldwide burden and epidemiological trends of tracheal, bronchus, and lung cancer: A population-based study - PubMed [Internet]. [cited 2025 Mar 7]. Available from: https://pubmed.ncbi.nlm.nih.gov/35313216/10.1016/j.ebiom.2022.103951 PMC8935504 35313216

[64] Goyal LD, Verma M, Garg P, Bhatt G. Variations in the patterns of tobacco usage among indian females - findings from the global adult tobacco survey India. BMC Womens Health. 2022 Nov 11;22(1):442. 36368987 10.1186/s12905-022-02014-3 PMC9652978

[65] Goel S, Walia D, Kumar R. The hidden crisis: Health impacts of tobacco and nicotine products on Indian women. J Family Med Prim Care. 2024;13(11):4751–4. doi: 10.4103/jfmpc.jfmpc_1741_24 39723003 PMC11668366

[66] Singh PK, Jain P, Singh N, Singh L, Kumar C, Yadav A, et al. Social desirability and under-reporting of smokeless tobacco use among reproductive age women: Evidence from National Family Health Survey. SSM Popul Health. 2022;19:101257. doi: 10.1016/j.ssmph.2022.101257 36263294 PMC9573902

[67] Tobacco Use and Effects of Professional Advice on Smoking Cessation among Youth in India - PubMed [Internet]. [cited 2025 Mar 7]. Available from: https://pubmed.ncbi.nlm.nih.gov/28749122/10.22034/APJCP.2017.18.7.1861 PMC5648391 28749122

[68] Dasgupta A, Yadav A, Paul B, Roy S, Ghosh P, Ghose S. Tobacco exposure is a menace among women: - A cross-sectional study in a rural area of West Bengal, India. J Family Med Prim Care. 2020;9(10):5288–94. doi: 10.4103/jfmpc.jfmpc_649_20 33409204 PMC7773084

[69] Singh S, Jain R, Joshi I, Chandra R, Singh L, Singh PK. Determinants of initiation, continuation and cessation of smokeless tobacco use among pregnant and lactating women: a qualitative study from low-income communities in urban India. Health Policy Plan. 2023 Sep 18;38(8):907–15. 37494416 10.1093/heapol/czad056

[70] Rodríguez-Bolaños R, Caballero M, Ponciano-Rodríguez G, González-Robledo LM, Cartujano-Barrera F, Reynales-Shigematsu LM, et al. Gender-related beliefs and attitudes about tobacco use and smoking cessation in Mexico. Health Psychol Behav Med. 2021 Jun 10;9(1):547–66. 34178431 10.1080/21642850.2021.1935963 PMC8204955

[71] The association between gender roles and smoking initiation among women and adolescent girls - PubMed [Internet]. [cited 2025 Mar 7]. Available from: https://pubmed.ncbi.nlm.nih.gov/33414576/10.1080/09589236.2019.1693985 PMC7787365 33414576

[72] Prevalence of Second Hand Smoke Exposure and Measures to Overcome: A Cross Sectional Study among Youth in Urban Hyderabad - PubMed [Internet]. [cited 2025 Mar 7]. Available from: https://pubmed.ncbi.nlm.nih.gov/39096250/10.4103/ijph.ijph_344_23 39096250

[73] Orton S, Jones LL, Cooper S, Lewis S, Coleman T. Predictors of children’s secondhand smoke exposure at home: a systematic review and narrative synthesis of the evidence. PLoS One. 2014;9(11):e112690. doi: 10.1371/journal.pone.0112690 25397875 PMC4232519

[74] Kim H, Kang H, Choi J, Cho S-I. Trends in adolescent secondhand smoke exposure at home over 15 years in Korea: Inequality by parental education level. Tob Induc Dis. 2023;21:88. doi: 10.18332/tid/166132 37396113 PMC10311469

[75] Chopra M, Gupta A, Sharma B, Kakade N, Arora M. Assessing second-hand smoke exposure among non-smoking youth in India: Insights from GATS I & II. Indian J Med Res. 2024;160(6):578–91. doi: 10.25259/IJMR_388_2024 39913523 PMC11801776

[76] Verma M, Kathirvel S, Das M, Aggarwal R, Goel S. Trends and patterns of second-hand smoke exposure amongst the non-smokers in India-A secondary data analysis from the Global Adult Tobacco Survey (GATS) I & II. PLoS One. 2020;15(6):e0233861. doi: 10.1371/journal.pone.0233861 32520979 PMC7286505

[77] Smoke-free status of homes and workplaces among Indian people: Evidence from Global Adult Tobacco SurveyData-2016/2017 - PubMed [Internet]. [cited 2025 Mar 7]. Available from: https://pubmed.ncbi.nlm.nih.gov/36821629/10.1371/journal.pone.0282138 PMC9949653 36821629

[78] Tobacco Control Policy in India: Progress and Challenges Quantified Using the Tobacco Control Scale - PubMed [Internet]. [cited 2025 Mar 7]. Available from: https://pubmed.ncbi.nlm.nih.gov/39342600/10.31557/APJCP.2024.25.9.3209 PMC11700346 39342600

[79] Kaur J, Prasad VM. Air nicotine monitoring for second hand smoke exposure in public places in India. Indian J Community Med. 2011;36(2):98–103. doi: 10.4103/0970-0218.84126 21976792 PMC3180954

[80] Banandur PS, Kumar MV, Gopalkrishna G. Awareness and compliance to anti-smoking law in South Bengaluru, India. Tob Prev Cessat. 2017;3:123. doi: 10.18332/tpc/76549 32432197 PMC7232790

[81] WHO report on the global tobacco epidemic, 2023: protect people from tobacco smoke [Internet]. [cited 2025 Mar 7]. Available from: https://www.who.int/publications/i/item/9789240077164

