# Supplementary material for: A comparative analysis of the burden, trends and inequalities of tracheal, bronchus, and lung cancer in India from 2000 to 2021: A systematic analysis for the Global Burden of Disease study 2021
Source: PLoS One. 2025 May 7;20(5):e0322646. doi: 10.1371/journal.pone.0322646 (PMC12058026; doi:10.1371/journal.pone.0322646)
Supplement: S1 Table — (DOCX) [file pone.0322646.s001.docx]

S1 Table. The burden of TBL cancer deaths associated with tobacco exposure among male in India in 2000 and 2021 and the temporal trend from 2000 to 2021

| male | tobacco | | | | | | smoking | | | | | | secondhand smoke | | | | | |
| --- | --- | --- | --- | --- | --- | --- | --- | --- | --- | --- | --- | --- | --- | --- | --- | --- | --- | --- |
|  | 2000 | | 2021 | | 2000-2021 | | 2000 | | 2021 | | 2000-2021 | | 2000 | | 2021 | | 2000-2021 | |
|  | deaths case  (95% UI） | ASMR  (95% UI） | deaths case  (95% UI） | ASMR  (95% UI） | Relative difference(%) | AAPC  (95% CI） | deaths case  (95% UI） | ASMR  (95% UI） | deaths case  (95% UI） | ASMR  (95% UI） | Relative difference(%) | AAPC  (95% CI） | deaths case  (95% UI） | ASMR  (95% UI） | deaths case  (95% UI） | ASMR  (95% UI） | Relative difference(%) | AAPC  (95% UI） |
| India | 16041 (13850-18345) | 5.15 (4.42-5.88) | 29577 (22853-35625) | 5.19 (4.02-6.26) | 84.38 | 0.03 (-0.12-0.18) | 15686 (13636-17790) | 5.04 (4.37-5.74) | 28853 (22386-34673) | 5.06 (3.93-6.07) | 83.94 | 0.02 (-0.13-0.18) | 1013 (123-1948) | 0.33 (0.04-0.62) | 1789 (238-3662) | 0.31 (0.04-0.64) | 76.63 | -0.24 (-0.41--0.07) |
| Andhra Pradesh | 454 (299-644) | 2.99 (1.99-4.21) | 801 (495-1165) | 2.95 (1.83-4.28) | 76.37 | 0 (-0.42-0.42) | 446 (294-630) | 2.94 (1.96-4.13) | 787 (486-1145) | 2.90 (1.80-4.21) | 76.45 | -0.02 (-0.35-0.32) | 24 (2-58) | 0.16 (0.01-0.38) | 38 (4-93) | 0.14 (0.01-0.34) | 57.3 | -0.54 (-1.26-0.18) |
| Assam | 533 (412-655) | 7.63 (5.83-9.5) | 992 (706-1301) | 8.17 (5.82-10.7) | 86.04 | 0.42 (-0.49-1.34) | 520 (403-638) | 7.46 (5.72-9.26) | 966 (692-1262) | 7.97 (5.75-10.43) | 85.58 | 0.4 (-0.51-1.32) | 34 (4-75) | 0.49 (0.06-1.07) | 69 (8-156) | 0.55 (0.07-1.25) | 99.73 | 0.65 (0.3-1) |
| Bihar | 189 (139-251) | 0.82 (0.6-1.08) | 296 (202-408) | 0.69 (0.48-0.95) | 56.98 | -0.78 (-1.89-0.34) | 183 (135-241) | 0.79 (0.58-1.04) | 285 (196-390) | 0.67 (0.47-0.91) | 56.02 | -0.82 (-1.96-0.33) | 11 (1-24) | 0.05 (0.01-0.1) | 18 (2-40) | 0.04 (0-0.09) | 59 | -0.6 (-1.4-0.2) |
| Chhattisgarh | 205 (145-274) | 3.81 (2.68-5.11) | 452 (321-626) | 4.35 (3.1-5.93) | 121.01 | 0.6 (-0.49-1.7) | 201 (142-266) | 3.74 (2.62-5.01) | 441 (315-607) | 4.25 (3.06-5.78) | 120.07 | 0.57 (-0.53-1.69) | 8 (1-17) | 0.15 (0.02-0.32) | 19 (2-42) | 0.18 (0.02-0.4) | 128.19 | 0.83 (-0.18-1.84) |
| Delhi | 318 (255-384) | 9.47 (7.67-11.46) | 663 (419-908) | 8.04 (5.08-10.92) | 108.42 | -1.32(-2.02--0.61) | 308 (250-372) | 9.2 (7.5-11.03) | 644 (414-875) | 7.81 (4.99-10.61) | 108.73 | -1.33 (-2.04--0.61) | 25 (2-57) | 0.76 (0.07-1.71) | 42 (4-102) | 0.51 (0.05-1.23) | 65.12 | -1.84 (-2.96--0.71) |
| Goa | 7 (4-10) | 1.41 (0.95-2.02) | 13 (8-20) | 1.39 (0.86-2.13) | 96.34 | -0.05 (-0.43-0.32) | 6 (4-9) | 1.35 (0.92-1.93) | 13 (8-19) | 1.34 (0.84-2.03) | 97.58 | -0.03 (-0.41-0.35) | 0 (0-1) | 0.09 (0.01-0.19) | 1 (0-2) | 0.08 (0.01-0.18) | 66.05 | -0.67 (-1.16--0.19) |
| Gujarat | 735 (603-881) | 5.29 (4.35-6.33) | 1504 (1035-2007) | 5.29 (3.63-7.09) | 104.77 | 0.1 (-0.58-0.78) | 718 (595-857) | 5.18 (4.26-6.17) | 1466 (1015-1938) | 5.16 (3.57-6.87) | 104.13 | 0.08 (-0.59-0.76) | 43 (5-93) | 0.31 (0.03-0.67) | 89 (11-197) | 0.31 (0.04-0.69) | 107.86 | 0.11 (-0.17-0.39) |
| Haryana | 369 (302-450) | 5.41 (4.44-6.59) | 749 (518-1004) | 6.17 (4.27-8.21) | 102.78 | 0.61 (0.3-0.91) | 360 (296-438) | 5.29 (4.34-6.42) | 733 (509-982) | 6.04 (4.19-8.04) | 103.37 | 0.67 (0.34-1.01) | 30 (3-60) | 0.43 (0.05-0.87) | 52 (5-119) | 0.43 (0.04-0.96) | 75.61 | -0.02 (-0.35-0.32) |
| Himachal Pradesh | 141 (113-174) | 6.5 (5.2-8) | 329 (230-447) | 8.65 (6.01-11.7) | 132.89 | 1.31 (0.91-1.7) | 139 (112-171) | 6.41 (5.15-7.86) | 325 (227-440) | 8.54 (5.97-11.57) | 133.2 | 1.31 (0.92-1.71) | 6 (0-16) | 0.29 (0.03-0.71) | 13 (1-31) | 0.35 (0.04-0.81) | 107.43 | 0.72 (0.05-1.4) |
| Jharkhand | 63 (46-81) | 0.94 (0.69-1.22) | 68 (45-114) | 0.51 (0.34-0.84) | 7.94 | -3.16 (-4.41--1.9) | 61 (45-78) | 0.91 (0.67-1.17) | 66 (44-112) | 0.5 (0.33-0.83) | 8.38 | -3.15 (-4.44--1.84) | 3 (0-7) | 0.05 (0.01-0.09) | 3 (0-7) | 0.02 (0-0.05) | -4.29 | -3.2 (-3.75--2.64) |
| Karnataka | 720 (568-892) | 4.28 (3.35-5.34) | 1328 (919-1783) | 4.59 (3.16-6.1) | 84.39 | 0.28 (-0.29-0.85) | 700 (555-860) | 4.17 (3.3-5.17) | 1288 (901-1738) | 4.45 (3.10-5.96) | 84.05 | 0.27 (-0.32-0.86) | 51 (7-105) | 0.3 (0.04-0.62) | 87 (9-206) | 0.3 (0.03-0.71) | 70.97 | 0.01 (-0.5-0.52) |
| Kerala | 1988 (1677-2324) | 16.58 (13.92-19.52) | 2729 (2019-3557) | 13.07 (9.71-17.06) | 37.25 | -1.12 (-1.5--0.73) | 1957 (1658-2287) | 16.32 (13.81-19.11) | 2680 (1982-3489) | 12.83 (9.54-16.72) | 36.95 | -1.13 (-1.51--0.74) | 103 (10-233) | 0.87 (0.09-1.98) | 122 (16-282) | 0.59 (0.07-1.34) | 17.98 | -1.91 (-2.38--1.44) |
| Madhya Pradesh | 959 (728-1188) | 5.78 (4.36-7.23) | 1663 (1183-2253) | 5.38 (3.84-7.19) | 73.45 | -0.34 (-0.55--0.14) | 937 (717-1157) | 5.65 (4.29-6.99) | 1608 (1144-2175) | 5.2 (3.74-6.96) | 71.67 | -0.33 (-0.56--0.11) | 62 (7-142) | 0.38 (0.04-0.89) | 113 (12-259) | 0.37 (0.04-0.85) | 83.2 | -0.05 (-0.34-0.23) |
| Maharashtra | 1103 (904-1313) | 3.55 (2.87-4.23) | 1379 (997-1857) | 2.39 (1.73-3.2) | 25 | -1.93 (-2.55--1.31) | 1071 (886-1270) | 3.45 (2.83-4.09) | 1322 (978-1772) | 2.29 (1.69-3.06) | 23.5 | -1.99 (-2.6--1.38) | 69 (8-155) | 0.22 (0.03-0.49) | 93 (10-213) | 0.16 (0.02-0.36) | 34.2 | -1.62 (-1.95--1.28) |
| Manipur | 65 (44-91) | 11.09 (7.48-15.33) | 157 (106-220) | 12.72 (8.68-17.54) | 140.51 | 0.59 (0.24-0.93) | 64 (43-90) | 10.94 (7.34-15.07) | 155 (105-216) | 12.54 (8.61-17.28) | 140.44 | 0.59 (0.24-0.93) | 3 (0-7) | 0.55 (0.06-1.15) | 7 (1-15) | 0.57 (0.05-1.21) | 119.67 | 0.24 (-0.46-0.96) |
| Meghalaya | 56 (40-76) | 12.29 (8.82-16.5) | 130 (90-182) | 15.39 (10.79-21.48) | 133.95 | 1.14 (0.69-1.59) | 55 (40-75) | 12.14 (8.75-16.29) | 129 (89-180) | 15.2 (10.66-21.09) | 133.85 | 1.14 (0.69-1.59) | 3 (0-8) | 0.75 (0.07-1.72) | 8 (1-20) | 0.92 (0.11-2.32) | 131.38 | 0.98 (0.29-1.67) |
| Mizoram | 45 (32-65) | 21.39 (14.92-29.89) | 123 (84-182) | 28.64 (19.55-42) | 170.96 | 1.47 (0.8-2.14) | 45 (31-64) | 21.07 (14.69-29.33) | 122 (83-179) | 28.23 (19.40-41.39) | 171.07 | 1.47 (0.8-2.14) | 3 (0-8) | 1.66 (0.18-3.76) | 8 (1-21) | 2 (0.23-4.97) | 149.02 | 0.97 (0.5-1.45) |
| Nagaland | 23 (15-33) | 5.76 (3.86-8.01) | 34 (23-52) | 5.95 (3.91-8.95) | 48.19 | 0.18 (-0.34-0.7) | 23 (15-32) | 5.64 (3.8-7.76) | 34 (22-51) | 5.81 (3.84-8.71) | 47.85 | 0.16 (-0.35-0.68) | 1 (0-3) | 0.33 (0.04-0.79) | 2 (0-4) | 0.32 (0.04-0.73) | 34.43 | -0.19 (-0.6-0.22) |
| Odisha | 230 (176-292) | 1.91 (1.43-2.42) | 442 (311-605) | 1.94 (1.38-2.64) | 91.73 | 0.12 (-0.5-0.74) | 225 (171-285) | 1.86 (1.4-2.37) | 429 (308-586) | 1.88 (1.35-2.55) | 90.95 | 0.1 (-0.51-0.71) | 12 (1-25) | 0.1 (0.01-0.22) | 23 (3-56) | 0.1 (0.01-0.25) | 94.58 | 0.09 (-0.51-0.7) |
| Other Union Territories | 24 (15-34) | 3.4 (2.24-4.74) | 66 (42-96) | 4.1 (2.65-5.86) | 178.16 | 0.93 (0.25-1.61) | 23 (15-33) | 3.34 (2.2-4.65) | 64 (41-93) | 4.02 (2.61-5.78) | 178.2 | 0.92 (0.24-1.61) | 1 (0-3) | 0.17 (0.02-0.38) | 3 (0-8) | 0.19 (0.02-0.44) | 153.55 | 0.57 (0.12-1.01) |
| Punjab | 219 (171-278) | 2.45 (1.92-3.11) | 373 (257-510) | 2.27 (1.58-3.11) | 70.75 | -0.14 (-1.41-1.14) | 207 (166-258) | 2.32 (1.85-2.91) | 354 (247-483) | 2.16 (1.51-2.95) | 71.14 | -0.13 (-1.44-1.2) | 19 (2-41) | 0.21 (0.03-0.46) | 29 (3-63) | 0.17 (0.02-0.38) | 53.8 | -0.64 (-1.65-0.38) |
| Rajasthan | 710 (5523-884) | 4.58 (3.57-5.73) | 1730 (1245-2324) | 6.31 (4.57-8.41) | 143.58 | 1.84 (0.82-2.86) | 697 (546-864) | 4.5 (3.52-5.64) | 1697 (1229-2268) | 6.2 (4.52-8.22) | 143.66 | 1.84 (0.82-2.87) | 49 (7-103) | 0.31 (0.04-0.66) | 102 (11-243) | 0.37 (0.04-0.86) | 109.37 | 0.96 (0.09-1.83) |
| Sikkim | 8 (6-11) | 6.01 (4.24-7.96) | 13 (8-21) | 5.05 (3.16-7.89) | 70.51 | -0.82 (-1.25--0.39) | 8 (5-10) | 5.84 (4.16-7.71) | 13 (8-20) | 4.88 (3.08-7.58) | 69.5 | -0.85 (-1.28--0.42) | 1 (0-1) | 0.41 (0.04-0.93) | 1 (0-2) | 0.34 (0.04-0.9) | 71.2 | -0.83 (-1.28--0.38) |
| Tamil Nadu | 1307 (1047-1586) | 5.54 (4.45-6.73) | 2156 (1494-2891) | 5.26 (3.70-7.02) | 64.94 | -0.3 (-0.48--0.11) | 1275 (1031-1537) | 5.4 (4.35-6.52) | 2104 (1472-2821) | 5.13 (3.63-6.83) | 65.05 | -0.29 (-0.48--0.11) | 87 (10-186) | 0.37 (0.04-0.79) | 118  (11-271) | 0.29 (0.03-0.67) | 35.75 | -1.06 (-1.37--0.75) |
| Telangana | 310 (202-436) | 3.45 (2.29-4.78) | 708 (433-1036) | 4.59 (2.82-6.7) | 128.33 | 1.33 (0.8-1.87) | 304 (198-42) | 3.39 (2.25-4.68) | 697 (429-1024) | 4.53 (2.8-6.6) | 129.6 | 1.36 (0.82-1.89) | 13 (1-32) | 0.14 (0.01-0.33) | 25 (2-7) | 0.16 (0.02-0.35) | 85.82 | 0.63 (0.13-1.13) |
| Tripura | 108 (75-151) | 12.03(8.4-16.68) | 215 (146-302) | 13.04 (8.92-18.12) | 99.52 | 0.44 (-0.11-0.99) | 107 (74-148) | 11.9 (8.3-16.44) | 212 (145-299) | 12.88 (8.85-17.96) | 99.2 | 0.43 (-0.12-0.98) | 5 (1-13) | 0.58 (0.06-1.44) | 10 (1-23) | 0.6 (0.07-1.39) | 91.42 | 0.2 (-0.48-0.89) |
| Uttar Pradesh | 2118 (1655-2624) | 4.3 (3.34-5.37) | 4539 (3444-6006) | 5.43 (4.14-7.17) | 114.31 | 1.44 (1.21-1.67) | 2072 (1628-2547) | 4.21 (3.29-5.21) | 4416 (3360-5796) | 5.29 (4.04-6.93) | 113.12 | 1.41 (1.18-1.65) | 131 (15-285) | 0.26 (0.03-0.57) | 286 (35-662) | 0.34 (0.04-0.78) | 117.74 | 1.28 (0.91-1.65) |
| Uttarakhand | 267 (193-361) | 11.35 (8.31-15.13) | 704 (481-939) | 15.71 (10.77-20.6) | 163.67 | 1.67 (1.14-2.21) | 263 (191-357) | 11.18 (8.30-14.86) | 694 (475-922) | 15.49 (10.68-20.27) | 163.95 | 1.67 (1.13-2.21) | 15 (2-32) | 0.61 (0.07-1.34) | 35 (3-78) | 0.77 (0.07-1.69) | 140.45 | 1.09 (0.72-1.45) |
| West Bengal | 2458 (2000-2966) | 10.54 (8.49-12.66) | 4561 (3346-5927) | 9.76 (7.18-12.65) | 85.59 | -0.38 (-1.06-0.29) | 2411 (1973-2911) | 10.34 (8.39-12.4) | 4466 (3290-5825) | 9.56 (7.10-12.34) | 85.23 | -0.39 (-1.05-0.28) | 176 (21-368) | 0.77 (0.09-1.61) | 328 (42-739) | 0.71 (0.09-1.6) | 86.51 | -0.37 (-1.21-0.48) |
