# Supplementary material for: A comparative analysis of the burden, trends and inequalities of tracheal, bronchus, and lung cancer in India from 2000 to 2021: A systematic analysis for the Global Burden of Disease study 2021
Source: PLoS One. 2025 May 7;20(5):e0322646. doi: 10.1371/journal.pone.0322646 (PMC12058026; doi:10.1371/journal.pone.0322646)
Supplement: S2 Table — (DOCX) [file pone.0322646.s002.docx]

S2 Table. The burden of TBL cancer deaths associated with tobacco exposure among female in India in 2000 and 2021 and the temporal trend from 2000 to 2021

| female | tobacco | | | | | | smoking | | | | | | secondhand smoke | | | | | |
| --- | --- | --- | --- | --- | --- | --- | --- | --- | --- | --- | --- | --- | --- | --- | --- | --- | --- | --- |
|  | 2000 | | 2021 | | 2000-2021 | | 2000 | | 2021 | | 2000-2021 | | 2000 | | 2021 | | 2000-2021 | |
|  | deaths case  (95% UI） | ASMR  (95% UI） | deaths case  (95% UI） | ASMR  (95% UI） | Relative difference(%) | AAPC  (95% CI） | deaths case  (95% UI） | ASMR  (95% UI） | deaths case  (95% UI） | ASMR  (95% UI） | Relative difference(%) | AAPC  (95% CI） | deaths case  (95% UI） | ASMR  (95% UI） | deaths case  (95% UI） | ASMR  (95% UI） | Relative difference(%) | AAPC  (95% UI） |
| India | 1653 (1104-2225) | 0.56 (0.38-0.76) | 3970 (2641-5653) | 0.67 (0.44-0.94) | 140.18 | 0.78 (0.46-1.09) | 1269 (981-1619) | 0.44 (0.34-0.57) | 3101 (2281-4100) | 0.53 (0.39-0.69) | 144.43 | 0.81 (0.49-1.12) | 468 (62-895) | 0.32 (0.04-0.64) | 1027  (146-2117) | 0.17 (0.02-0.34) | 119.31 | 0.46 (0.16-0.76) |
| Andhra Pradesh | 68 (41-104) | 0.43 (0.26-0.65) | 143 (81-217) | 0.45 (0.26-0.69) | 111.03 | 0.31 (-0.04-0.66) | 59 (36-90) | 0.37 (0.23-0.55) | 124 (75-186) | 0.39 (0.24-0.59) | 110.22 | 0.3 (-0.05-0.65) | 12 (1-28) | 0.08 (0.01-0.18) | 24 (3-57) | 0.08 (0.01-0.18) | 99.09 | 0.04 (-0.33-0.42) |
| Assam | 55 (37-77) | 0.95 (0.64-1.34) | 118 (68-186) | 1 (0.57-1.57) | 114.75 | 0.27 (-0.11-0.65) | 46 (32-61) | 0.8 (0.56-1.08) | 90 (57-130) | 0.78 (0.5-1.11) | 97.57 | -0.16 (-0.58-0.26) | 12 (2-27) | 0.19 (0.02-0.42) | 33 (5-78) | 0.27 (0.04-0.62) | 182.37 | 1.69 (1.02-2.37) |
| Bihar | 63 (44-86) | 0.31 (0.21-0.43) | 147 (102-210) | 0.38 (0.27-0.54) | 135.06 | 0.98 (0.26-1.71) | 59 (42-81) | 0.3 (0.21-0.41) | 140 (99-201) | 0.36 (0.25-0.52) | 136.15 | 0.99 (0.27-1.73) | 5 (1-10) | 0.02 (0-0.05) | 10 (1-22) | 0.02 (0-0.05) | 97.54 | 0.25 (-0.32-0.81) |
| Chhattisgarh | 11 (6-18) | 0.19 (0.1-0.29) | 27 (14-44) | 0.23 (0.12-0.39) | 135.92 | 1.19 (0.32-2.06) | 9 (5-14) | 0.15 (0.08-0.23) | 21 (12-34) | 0.18 (0.11-0.31) | 125.95 | 1.01 (0.13-1.89) | 3 (0-5) | 0.04 (0-0.08) | 7 (1-15) | 0.06 (0.01-0.12) | 165.58 | 1.81 (0.89-2.73) |
| Delhi | 29 (18-44) | 1 (0.62-1.51) | 61 (36-95) | 0.73 (0.43-1.14) | 107.02 | -1.51 (-2.34--0.68) | 22 (15-32) | 0.76 (0.53-1.14) | 47 (32-70) | 0.57 (0.37-0.83) | 115.28 | -1.37 (-2.22--0.52) | 9 (1-20) | 0.3 (0.03-0.67) | 15 (2-35) | 0.19 (0.02-0.42) | 69.72 | -2.32 (-3--1.63) |
| Goa | 2 (1-3) | 0.37 (0.2-0.59) | 4 (2-7) | 0.35 (0.2-0.58) | 115.29 | -0.18 (-0.42-0.05) | 2 (1-3) | 0.33 (0.18-0.52) | 4 (2-6) | 0.31 (0.17-0.5) | 116.22 | -0.24 (-0.47--0.01) | 0 (0-1) | 0.05 (0.01-0.1) | 1 (0-1) | 0.05 (0.01-0.11) | 104.4 | -0.01 (-0.51-0.49) |
| Gujarat | 54 (34-79) | 0.4 (0.26-0.57) | 188 (113-287) | 0.6 (0.36-0.9) | 247.96 | 2.1 (0.89-3.32) | 41 (28-56) | 0.32 (0.21-0.43) | 141 (92-97) | 0.46 (0.3-0.65) | 246.37 | 2.01 (0.91-3.12) | 16 (2-33) | 0.1 (0.01-0.21) | 56 (6-124) | 0.17 (0.02-0.37) | 248.64 | 2.54 (2.23-2.86) |
| Haryana | 49 (32-69) | 0.84 (0.57-1.2) | 148 (90-227) | 1.21 (0.75-1.86) | 202.38 | 1.85 (1.4-2.3) | 38 (28-55) | 0.68 (0.48-0.96) | 119 (76-175) | 0.98 (0.63-1.45) | 208.66 | 1.94 (1.22-2.65) | 14 (2-28) | 0.22 (0.03-0.47) | 37 (4-85) | 0.3 (0.03-0.67) | 171.72 | 1.35 (1.04-1.67) |
| Himachal Pradesh | 12 (8-17) | 0.6 (0.4-0.87) | 28 (18-41) | 0.63 (0.4-0.94) | 134.88 | 0.42 (-0.38-1.23) | 10 (7-14) | 0.51 (0.35-0.73) | 23 (15-34) | 0.54 (0.35-0.78) | 137.98 | 0.39 (-0.51-1.3) | 2 (0-5) | 0.11 (0.01-0.24) | 5 (1-12) | 0.12 (0.01-0.27) | 118.63 | 0.39 (-1.07-1.86) |
| Jharkhand | 11 (6-17) | 0.18 (0.1-0.28) | 26 (15-43) | 0.2 (0.11-0.32) | 136.72 | 0.32 (-1.02-1.67) | 9 (5-14) | 0.15 (0.09-0.25) | 22 (13-36) | 0.17 (0.1-0.27) | 134.7 | 0.23 (-1.13-1.61) | 2 (0-3) | 0.44 (0.05-0.96) | 4 (1-9) | 0.03 (0-0.07) | 146.03 | 0.79 (-0.62-2.23) |
| Karnataka | 62 (34-96) | 0.35 (0.2-0.55) | 148 (75-249) | 0.44 (0.22-0.74) | 138.61 | 1.11 (0.77-1.44) | 41 (26-59) | 0.23 (0.15-0.34) | 96 (55-141) | 0.28 (0.16-0.42) | 136.12 | 0.92 (0.34-1.5) | 24 (3-49) | 0.03 (0-0.05) | 56 (7-129) | 0.17 (0.02-0.38) | 134.5 | 1.09 (0.77-1.42) |
| Kerala | 93 (52-149) | 0.65 (0.36-1.06) | 130 (68-204) | 0.49 (0.26-0.77) | 39.99 | -1.39 (-1.98--0.8) | 62 (38-95) | 0.44 (0.27-0.66) | 92 (53-138) | 0.34 (0.2-0.52) | 46.77 | -1.2 (-1.86--0.53) | 35 (4-74) | 0.13 (0.02-0.27) | 42 (5-93) | 0.16 (0.02-0.36) | 19.59 | -1.99 (-2.2--1.78) |
| Madhya Pradesh | 81 (42-132) | 0.5 (0.26-0.8) | 160 (82-280) | 0.47 (0.24-0.81) | 97.43 | -0.33 (-0.87-0.21) | 51 (32-74) | 0.32 (0.2-0.47) | 102 (62-159) | 0.3 (0.18-0.46) | 99.74 | -0.22 (-0.61-0.18) | 34 (4-78) | 0.24 (0.03-0.51) | 63 (7-147) | 0.18 (0.02-0.42) | 85.56 | -0.5 (-1.03-0.03) |
| Maharashtra | 104 (57-162) | 0.33 (0.18-0.51) | 199 (107-343) | 0.32 (0.17-0.54) | 92.03 | -0.18 (-0.71-0.35) | 69 (43-105) | 0.22 (0.14-0.34) | 136 (83-217) | 0.22 (0.13-0.34) | 97.03 | -0.03 (-0.73-0.68) | 38 (5-81) | 0.2 (0.02-0.47) | 68 (8-153) | 0.11 (0.01-0.24) | 76.27 | -0.53 (-1.05-0) |
| Manipur | 26 (16-37) | 4.6 (2.85-6.55) | 62 (41-89) | 4.72 (3.13-6.77) | 140.6 | 0.1 (-0.33-0.52) | 24 (15-34) | 4.35 (2.65-6.18) | 58 (38-81) | 4.44 (2.95-6.27) | 138.73 | 0.06 (-0.36-0.48) | 3 (0-6) | 0.12 (0.02-0.25) | 6 (1-14) | 0.45 (0.05-0.97) | 138.86 | 0.06 (-0.13-0.25) |
| Meghalaya | 6 (3-10) | 1.51 (0.75-2.41) | 16 (9-25) | 1.66 (0.96-2.64) | 154.44 | 0.43 (-0.16-1.03) | 5 (3-7) | 1.19  (0.68-1.85) | 12 (7-17) | 1.24 (0.76-1.84) | 141.06 | 0.14 (-0.29-0.57) | 2 (0-5) | 0.45 (0.05-0.94) | 5 (1-12) | 0.53 (0.07-1.19) | 182.17 | 1.06 (0.49-1.63) |
| Mizoram | 23 (15-33) | 11.47 (7.46-16.78) | 67 (44-98) | 14.48 (9.63-20.95) | 196.13 | 1.08 (0.62-1.54) | 21 (14-30) | 10.55 (6.97-15.3) | 61 (41-87) | 13.18 (8.87-18.58) | 193.48 | 1.04 (0.58-1.49) | 4 (0-8) | 0.43 (0.04-1.02) | 11 (1-24) | 2.29 (0.25-4.98) | 201.79 | 1.26 (0.98-1.54) |
| Nagaland | 2 (1-3) | 0.49 (0.24-0.83) | 3 (1-4) | 0.46 (0.23-0.73) | 59.52 | -0.3 (-0.8-0.21) | 1 (1-2) | 0.33 (0.19-0.52) | 2 (1-3) | 0.31 (0.18-0.49) | 60.56 | -0.27 (-0.81-0.27) | 1 (0-1) | 1.79 (0.17-3.93) | 1 (0-2) | 0.16 (0.02-0.36) | 52.56 | -0.36 (-0.59--0.13) |
| Odisha | 23 (13-36) | 0.19 (0.1-0.3) | 58 (30-90) | 0.24 (0.13-0.38) | 149.04 | 1.17 (-0.23-2.58) | 16 (10-25) | 0.13 (0.08-0.2) | 42 (25-66) | 0.17 (0.1-0.27) | 155.92 | 1.28 (-0.56-3.17) | 8 (1-17) | 0.18 (0.02-0.43) | 17 (2-39) | 0.07 (0.01-0.16) | 128.92 | 0.96 (-0.03-1.97) |
| Other Union Territories | 2 (1-3) | 0.29 (0.14-0.49) | 6 (3-11) | 0.38 (0.17-0.65) | 220.6 | 1.22 (0.92-1.53) | 1 (1-2) | 0.18 (0.1-0.29) | 4 (2-6) | 0.23 (0.13-0.4) | 234.41 | 1.33 (0.91-1.76) | 1 (0-2) | 0.06 (0.01-0.14) | 3 (0-6) | 0.15 (0.02-0.36) | 201.13 | 0.92 (0.6-1.24) |
| Punjab | 21 (12-33) | 0.27 (0.15-0.42) | 37 (19-64) | 0.23 (0.12-0.4) | 72.89 | -0.77 (-1.37--0.17) | 14 (8-21) | 0.18 (0.11-0.29) | 24 (14-40) | 0.16 (0.09-0.25) | 74.95 | -0.75 (-1.46--0.04) | 8 (1-16) | 0.12 (0.01-0.27) | 13 (2-30) | 0.08 (0.01-0.18) | 66.25 | -0.95 (-1.36--0.54) |
| Rajasthan | 112 (74-162) | 0.74 (0.49-1.08) | 265 (158-420) | 0.88 (0.53-1.38) | 135.79 | 0.78 (0.36-1.2) | 94 (66-129) | 0.63 (0.43-0.88) | 216 (143-321) | 0.72 (0.49-1.09) | 129.88 | 0.65 (0.24-1.07) | 27 (4-56) | 0.09 (0.01-0.2) | 63 (7-146) | 0.2 (0.02-0.47) | 130.75 | 0.72 (0.36-1.09) |
| Sikkim | 3 (2-5) | 3.04 (2.04-4.42) | 8 (5-12) | 3.19 (2.08-5.03) | 155.73 | 0.17 (-0.2-0.53) | 3 (2-4) | 2.8 (1.88-3.97) | 7 (5-11) | 2.91 (1.86-4.47) | 154.32 | 0.13 (-0.23-0.49) | 0 (0-1) | 0.17 (0.02-0.35) | 1 (0-2) | 0.38 (0.05-0.93) | 143.59 | 0.01 (-0.43-0.44) |
| Tamil Nadu | 55 (26-97) | 0.25 (0.12-0.43) | 91 (46-153) | 0.21 (0.11-0.36) | 64.86 | -0.85 (-1.54--0.17) | 28 (17-45) | 0.13 (0.08-0.21) | 54 (33-84) | 0.13 (0.08-0.2) | 90.56 | -0.18 (-0.92-0.57) | 28 (3-64) | 0.38 (0.04-0.81) | 39 (4-85) | 0.09 (0.01-0.19) | 35.5 | -1.66 (-2.18--1.14) |
| Telangana | 30 (18-48) | 0.35 (0.21-0.55) | 108 (63-160) | 0.63 (0.36-0.93) | 255.97 | 2.8 (2.22-3.38) | 26 (16-42) | 0.31 (0.19-0.48) | 95 (57-137) | 0.56 (0.33-0.82) | 258.83 | 2.79 (2.16-3.41) | 5 (0-11) | 0.12 (0.01-0.28) | 16 (2-38) | 0.09 (0.01-0.2) | 246.86 | 2.94 (2.51-3.37) |
| Tripura | 11 (7-17) | 1.28 (0.81-1.96) | 22 (13-32) | 1.26 (0.77-1.87) | 99.3 | -0.04 (-0.49-0.42) | 10 (6-15) | 1.16 (0.75-1.7) | 19 (12-28) | 1.12 (0.73-1.65) | 98 | -0.1 (-0.54-0.35) | 2 (0-4) | 0.05 (0-0.11) | 4 (0-8) | 0.2 (0.02-0.42) | 82.64 | -0.31 (-0.82-0.2) |
| Uttar Pradesh | 338 (215-486) | 0.82 (0.52-1.16) | 912 (573-1405) | 1.1 (0.7-1.68) | 170.05 | 1.42 (0.3-2.55) | 278 (194-378) | 0.69 (0.47-0.94) | 762 (515-1101) | 0.93 (0.63-1.35) | 174.63 | 1.45 (0.26-2.66) | 79 (9-77) | 0.21 (0.02-0.5) | 188 (23-488) | 0.22 (0.03-0.56) | 137.96 | 1.06 (0.39-1.74) |
| Uttarakhand | 46 (29-66) | 2.04 (1.3-2.97) | 160 (102-232) | 3.29 (2.12-4.74) | 250.5 | 2.25 (1.72-2.79) | 38 (25-53) | 1.77 (1.17-2.49) | 138 (92-195) | 2.88 (1.96-4.08) | 259.53 | 2.31 (1.78-2.86) | 10 (1-21) | 0.18 (0.02-0.4) | 30 (3-66) | 0.58 (0.06-1.28) | 203.98 | 1.82 (1.38-2.26) |
| West Bengal | 202 (116-293) | 0.96 (0.58-1.4) | 477 (270-757) | 1.11 (0.64-1.76) | 136.36 | 0.68 (0.38-0.99) | 140 (97-190) | 0.7 (0.49-0.96) | 326 (214-482) | 0.79 (0.51-1.14) | 132.64 | 0.5 (0.22-0.78) | 74 (10-150) | 0.4 (0.04-0.84) | 175 (20-357) | 0.38 (0.04-0.79) | 135.87 | 0.8 (0.39-1.2) |
