# Supplementary material for: A comparative analysis of the burden, trends and inequalities of tracheal, bronchus, and lung cancer in India from 2000 to 2021: A systematic analysis for the Global Burden of Disease study 2021
Source: PLoS One. 2025 May 7;20(5):e0322646. doi: 10.1371/journal.pone.0322646 (PMC12058026; doi:10.1371/journal.pone.0322646)
Supplement: S3 Table — (DOCX) [file pone.0322646.s003.docx]

S3 Table. The burden of TBL cancer DALYs associated with tobacco exposure among male in India in 2000 and 2021 and the temporal trend from 2000 to 2021

| male | tobacco | | | | | | smoking | | | | | | secondhand smoke | | | | | |
| --- | --- | --- | --- | --- | --- | --- | --- | --- | --- | --- | --- | --- | --- | --- | --- | --- | --- | --- |
|  | 2000 | | 2021 | | 2000-2021 | | 2000 | | 2021 | | 2000-2021 | | 2000 | | 2021 | | 2000-2021 | |
|  | DALYs case  (95% UI） | ASDR  (95% UI） | DALYs case  (95% UI） | ASDR  (95% UI） | Relative difference(%) | AAPC  (95% CI） | DALYs case  (95% UI） | ASDR  (95% UI） | DALYs case  (95% UI） | ASDR  (95% UI） | Relative difference(%) | AAPC  (95% CI） | DALYs case  (95% UI） | ASDR  (95% UI） | DALYs case  (95% UI） | ASDR  (95% UI） | Relative difference(%) | AAPC  (95% UI） |
| India | 453421 (390081-519573) | 133.73 (115.1-153.18) | 787476 (603653-953392) | 128.76 (98.97-155.49) | 73.67 | -0.14 (-0.5-0.23) | 442402 (385246-501555) | 130.67 (113.76-148.39) | 766124 (593415-926257) | 125.41 (97.28-151.07) | 73.17 | -0.15 (-0.5-0.2) | 29107 (3513-56615) | 8.49 (1.03-16.41) | 49374.78 (65990-101621) | 7.97 (1.06-16.33) | 69.63 | -0.38 (-0.84-0.07) |
| Andhra Pradesh | 12845 (8498-18168) | 77.17 (50.53-109.03) | 20258 (12280-9858) | 73.04 (44.4-107.47) | 57.71 | -0.21 (-0.44-0.02) | 12577 (8272-17725) | 75.7 (49.58-106.85) | 19846 (12096-29259) | 71.59 (43.64-105.37) | 57.8 | -0.22 (-0.45-0.02) | 714 (60-1723) | 4.2 (0.35-10.11) | 1018 (94-2439) | 3.64 (0.33-8.69) | 42.67 | -0.65 (-1--0.3) |
| Assam | 15505 (12014-19072) | 201.64 (156.38-247.74) | 26990 (19096-35532) | 200.91 (142.76-263.85) | 74.07 | 0.14 (-0.69-0.98) | 15106 (11911-18499) | 196.79 (152.41-242.01) | 26219 (18645-34605) | 195.53 (139.92-255.92) | 73.57 | 0.03 (-0.54-0.6) | 1014 (113-2218) | 13.01 (1.47-28.5) | 1921 (232-4397) | 13.99 (1.69-31.91) | 89.44 | 0.43 (0.14-0.71) |
| Bihar | 5176 (3798-6861) | 20.64 (15.29-27.28) | 7365 (4957-10220) | 16.32 (11.07-22.47) | 42.3 | -1.11 (-2.22-0) | 4979 (3736-6596) | 19.91 (14.88-26.3) | 7035 (4759-9747) | 15.62 (10.67-21.41) | 41.28 | -1.16 (-2.3-0) | 331 (39-694) | 1.27 (0.15-2.68) | 486 (50-1074) | 1.05 (0.11-2.31) | 46.91 | -0.91 (-1.76--0.05) |
| Chhattisgarh | 5680 (4032-7604) | 93.37 (66.08-124.95) | 12058 (8492-16780) | 103.72 (73.57-142.93) | 112.27 | 0.58 (-0.25-1.42) | 5554 (3935-7387) | 91.49 (64.48-121.89) | 11726 (8304-16264) | 101.13 (72.83-138.07) | 111.13 | 0.55 (-0.28-1.4) | 242 (33-503) | 3.79 (0.5-7.99) | 541 (48-1235) | 4.42 (0.41-0.07) | 124.82 | 0.82 (-0.07-1.71) |
| Delhi | 9572 (7697-11578) | 248.12 (198.67-298.94) | 18070 (11498-24591) | 201.62 (128.79-274.23) | 88.78 | -1.12 (-2.77-0.56) | 9274 (7559-11128) | 240.77 (194.85-290.49) | 17545 (11286-23757) | 195.8 (126.49-265.17) | 89.2 | -1.12 (-2.78-0.57) | 763 (66-1701) | 19.81 (1.77-44.17) | 1153 (121-2796) | 12.85 (1.33-31.23) | 51.08 | -2.27 (-3.71--0.81) |
| Goa | 184 (120-266) | 35.66 (23.78-51.47) | 323 (196-499) | 33.71 (20.36-51.96) | 75.51 | -0.24 (-1.02-0.54) | 176 (115-252) | 34.1 (22.69-48.9) | 311 (191-474) | 32.36 (20-49.33) | 76.95 | -0.22 (-1.01-0.57) | 13 (1-29) | 2.33 (0.27-5.22) | 19 (2-43) | 1.94 (0.24-4.47) | 45.75 | -0.9 (-1.39--0.41) |
| Gujarat | 20767 (16923-25120) | 134.73 (109.86-162.28) | 40557 (27980-54171) | 129.63 (89.22-172.53) | 95.29 | -0.04 (-0.91-0.84) | 20262 (16654-24409) | 131.68 (108.47-158.28) | 39398 (27429-52172) | 126.13 (87.86-166.71) | 94.44 | -0.06 (-0.92-0.8) | 1224 (138-2607) | 7.82 (0.88-16.85) | 2488 (306-5337) | 7.82 (0.96-16.94) | 103.35 | 0.21 (-0.16-0.58) |
| Haryana | 10637 (8617-13015) | 149.33 (121.36-183.1) | 20604 (14322-27847) | 158.15 (109.65-213.14) | 93.69 | 0.38 (0-0.77) | 10350 (8489-12696) | 145.68 (120.16-178.19) | 20092 (14011-27063) | 154.51 (107.43-208.02) | 94.12 | 0.39 (0.01-0.78) | 859 (95-1728) | 11.78 (1.3-23.81) | 1492 (143-3395) | 11.22 (1.08-25.45) | 73.76 | -0.23 (-0.45-0) |
| Himachal Pradesh | 3775 (3009-4688) | 166.58 (132.93-206.82) | 8706 (6037-11750) | 215.53 (149.69-292.05) | 130.62 | 1.24 (0.92-1.55) | 3709 (2964-4596) | 163.84 (131.49-203.12) | 8563 (5970-11576) | 212.14 (148.15-287.03) | 130.87 | 1.24 (0.93-1.55) | 179 (17-434) | 7.77 (0.74-18.79) | 376 (40-882) | 9.17 (0.97-21.54) | 109.85 | 0.71 (0.38-1.04) |
| Jharkhand | 1764 (1295-2321) | 23.4 (17.26-30.46) | 1688 (1103-2911) | 11.44 (7.49-19.46) | -4.32 | -3.82 (-5.12--2.51) | 1694 (1262-2192) | 22.58 (16.98-28.98) | 1627  (1096-2839) | 11.05 (7.39-19.01) | -3.96 | -3.81 (-5.16--2.45) | 102 (12-212) | 1.24 (0.15-2.52) | 88 (11-184) | 0.57 (0.07-1.2) | -13.53 | -3.93 (-4.85--3.01) |
| Karnataka | 20724 (16353-25648) | 111.87 (88.19-138.89) | 34288 (23651-46343) | 109.57 (75.82-147.22) | 65.45 | -0.04 (-0.25-0.16) | 20075 (15933-24612) | 108.59 (86.25-133.85) | 33146 (22867-44537) | 106.07 (73.44-142.81) | 65.11 | -0.05 (-0.25-0.16) | 1498 (201-3081) | 7.99 (1.07-16.39) | 2339 (249-5482) | 7.37 (0.79-17.37) | 56.1 | -0.39 (-0.8-0.02) |
| Kerala | 53088 (44553-62074) | 416.56 (349.39-488.88) | 68573 (50136-90373) | 310.73 (228.1-407.46) | 29.17 | -1.23 (-1.54--0.92) | 52223 (44051-61045) | 409.92 (345.37-480.44) | 67285 (49507-88463) | 304.8 (225.02-399.04) | 28.84 | -1.24 (-1.55--0.92) | 2708 (267-6015) | 21.33 (2.11-47.55) | 3103 (399-7160) | 14.18 (1.81-32.09) | 14.58 | -1.96 (-2.26--1.66) |
| Madhya Pradesh | 27548 (20896-34145) | 150.68 (114.59-186.92) | 45172 (31887-62239) | 132.15 (94.06-180.09) | 63.98 | -0.63 (-0.98--0.28) | 26869 (20572-33029) | 147.1 (112.48-181.42) | 43554 (30694-59636) | 127.6 (90.41-173.22) | 62.1 | -0.64 (-0.88--0.39) | 1764 (200-4049) | 9.68 (1.08-22.15) | 3147 (335-7303) | 9.1 (0.96-21.08) | 78.42 | -0.28 (-0.51--0.04) |
| Maharashtra | 30465 (24987-36120) | 90.08 (73.87-107.1) | 34534 (24798-47176) | 56.97 (40.97-77.5) | 13.36 | -2.16 (-2.77--1.55) | 29464 (24301-34690) | 87.3 (72.05-103.03) | 32915 (24278-44352) | 54.4 (40.09-72.99) | 11.71 | -2.23 (-2.81--1.65) | 2009 (221-4428) | 5.76 (0.64-12.81) | 2535 (285-5856) | 4.07 (0.46-9.43) | 26.22 | -1.62 (-1.93--1.31) |
| Manipur | 1682 (1105-2372) | 262.59 (174.72-365.88) | 4016 (2681-5699) | 292.38 (197.87-410) | 138.83 | 0.51 (0.15-0.88) | 1652 (1089-2308) | 258.35 (171.09-358.51) | 3945 (2654-5562) | 287.64 (195.7-402.36) | 138.84 | 0.58 (-0.41-1.57) | 88 (10-186) | 13.43 (1.57-28.48) | 193 (18-406) | 13.69 (1.28-28.55) | 119.32 | 0.14 (-0.64-0.92) |
| Meghalaya | 1546 (1100-2134) | 298.87 (214.38-408.58) | 3420 (2315-4855) | 360.04 (246.51-506.23) | 121.21 | 0.99 (0.09-1.9) | 1525.24 (1086-2099) | 295.12 (212.81-403.32) | 3373 (2303-4743) | 355.35 (245.11-499.15) | 121.12 | 0.99 (0.08-1.91) | 93 (9-210) | 18.1 (1.75-41.04) | 205 (23-506) | 21.56 (2.5-53.9) | 121.03 | 0.84 (0.02-1.68) |
| Mizoram | 1302 (908-1881) | 545.35 (377.92-781.57) | 3252 (2189-4817) | 684.12 (463.74-1012.16) | 149.73 | 1.19 (0.32-2.07) | 1284 (893-1838) | 537.61 (373.06-769.03) | 3207 (2158-4759) | 674.67 (460.54-1000.06) | 149.86 | 1.2 (0.32-2.08) | 90 (9-207) | 39.66 (4.13-90.44) | 209 (23-540) | 45.3 (34.99-115.07) | 131.5 | 0.61 (0.27-0.95) |
| Nagaland | 633 (420-892) | 136.58 (90.49-191.49) | 858 (558-1322) | 138.34 (90.07-212.67) | 35.49 | 0.09 (-0.71-0.91) | 618 (409-863) | 133.53 (89.74-187.57) | 836 (547-1287) | 134.89 (88.63-208.71) | 35.22 | 0.08 (-0.73-0.9) | 40 (4-94) | 8.28 (0.9-19.73) | 49 (6-114) | 7.76 (0.91-17.99) | 23.89 | -0.28 (-1.06-0.51) |
| Odisha | 6734 (5204-8460) | 51.82 (39.64-65.68) | 11949 (8467-16357) | 49.99 (35.56-68.32) | 77.43 | -0.22 (-0.68-0.24) | 6557 (5111-8296) | 50.49 (39.12-64.55) | 11582 (8315-15802) | 48.45 (34.91-66.03) | 76.64 | -0.24 (-0.7-0.22) | 347 (40-742) | 2.67 (0.3-5.71) | 64 (68-1519) | 2.67 (0.29-6.42) | 82.76 | -0.01 (-0.49-0.47) |
| Other Union Territories | 667 (435-958) | 84 (54.73-119.39) | 1775 (1119-2630) | 99.19 (63.14-145.08) | 166.06 | 0.86 (0.04-1.68) | 649 (423-937) | 82.14 (53.86-116.34) | 1728 (1100-2549) | 96.86 (62.14-141.44) | 166.36 | 0.85 (0.03-1.68) | 39 (4-92) | 4.5 (0.49-10.26) | 95 (11-224) | 4.99 (0.58-11.71) | 142.11 | 0.55 (0.1-1) |
| Punjab | 5990 (4651-7647) | 64.5 (50.16-82.62) | 9734 (6616-13156) | 57.65 (39.31-77.84) | 62.49 | -0.68 (-0.94--0.42) | 5648 (4484-7034) | 60.94 (48.46-76.18) | 9210 (6386-12353) | 54.59 (38.11-73.11) | 63.07 | -0.57 (-1.09--0.04) | 531 (68-1167) | 5.61 (0.72-12.38) | 775 (88-1727) | 4.55 (0.52-10.1) | 45.91 | -0.97 (-1.44--0.5) |
| Rajasthan | 20469 (15864-25551) | 120.56 (93.5-150.49) | 48346 (34358-65400) | 161.48 (115.61-218.06) | 136.19 | 1.62 (0.74-2.5) | 20013 (15627-24862) | 118.16 (92.35-146.7) | 47261 (33959-63566) | 158.24 (114.24-212.4) | 136.15 | 1.61 (0.73-2.51) | 1440 (196-3031) | 8.33 (1.13-17.58) | 3008 (327-7230) | 9.72 (1.06-23.16) | 108.91 | 0.78 (-0.12-1.68) |
| Sikkim | 212 (148-288) | 144.04 (101.11-193.71) | 323 (199-510) | 111.72 (69.36-175.7) | 52.49 | -1.24 (-1.84--0.63) | 205 (144-277) | 139.86 (98.91-187.85) | 311 (193-489) | 107.64 (67.51-168.18) | 51.29 | -1.28 (-1.88--0.67) | 15 (2-33) | 9.95 (1.01-22.05) | 24 (3-65) | 7.96 (0.97-21.28) | 58.06 | -0.99 (-1.42--0.55) |
| Tamil Nadu | 37750 (30306-45574) | 148.94 (119.66-180.74) | 58061 (40185-8509) | 131.26 (91.13-175.95) | 53.8 | -0.7 (-0.86--0.55) | 36782 (29651-4340) | 145.17 (117.5-174.92) | 56585 (39342-75877) | 127.92 (89.61-170.72) | 53.84 | -0.53 (-0.94--0.11) | 2514 (291-5413) | 9.91 (1.15-21.36) | 3257 (315-7403) | 7.36 (0.72-16.73) | 29.58 | -1.4 (-1.64--1.15) |
| Telangana | 8700 (5661-12215) | 87.25 (57.12-121.97) | 18058 (10762-26854) | 106.78 (63.87-156.79) | 107.56 | 0.98 (0.68-1.27) | 8490 (5540-11931) | 85.4 (55.72-119.38) | 17724 (10660-26492) | 104.98 (63.52-154.57) | 108.77 | 1 (0.69-1.3) | 421 (39-987) | 3.91 (0.36-9.21) | 733 (70-1700) | 4.13 (0.4-9.47) | 74.01 | 0.27 (-0.22-0.76) |
| Tripura | 2940 (2027-4096) | 297.44 (204.81-417.14) | 5594 (3726-7928) | 311.76 (208.99-439.34) | 90.24 | 0.33 (-0.35-1.01) | 2902 (2011-4036) | 293.9 (202.3-411.76) | 5512 (3680-7789) | 307.5 (207.56-434.49) | 89.93 | 0.32 (-0.36-0.99) | 140 (15-345) | 14.18 (1.48-35.01) | 260 (29-606) | 14.34 (1.59-33.44) | 85.03 | 0.18 (-0.61-0.98) |
| Uttar Pradesh | 60099 (47579-74275) | 113.77 (89.46-141.52) | 124323 (93867-165302) | 138.94 (105.02-185.08) | 106.86 | 0.96 (0.46-1.46) | 58589 (46862-71866) | 111.13 (88.31-136.59) | 120518 (90631-158433) | 134.94 (101.48-177.41) | 105.7 | 0.93 (0.43-1.43) | 3880 (435-8347) | 7.19 (0.81-15.54) | 8190 (1001-18580) | 8.95 (1.1-20.3) | 111.07 | 1.08 (0.26-1.91) |
| Uttarakhand | 7999 (5725-10958) | 304.99 (222.12-413.85) | 19907 (13482-26791) | 404.86 (276.44-539.25) | 148.88 | 1.41 (0.84-1.97) | 7850 (5654-10696) | 299.79 (220.16-405.18) | 19555 (13291-26376) | 398.26 (272-531.54) | 149.12 | 1.41 (0.84-1.98) | 449 (50-988) | 16.81 (1.88-36.81) | 1032 (83-2322) | 20.55 (1.68-45.25) | 129.64 | 0.92 (0.61-1.23) |
| West Bengal | 70086 (57201-84713) | 273.13 (221.93-330.12) | 121030 (88335-157734) | 237.42 (173.49-308.85) | 72.69 | -0.66 (-1.16--0.16) | 68700 (56780-83104) | 267.97 (219.94-323.98) | 118323 (86097-154507) | 232.22 (170.31-302.52) | 72.23 | -0.67 (-1.19--0.16) | 4916 (589-10260) | 19.41 (2.33-40.68) | 8704 (1101-19446) | 17.16 (2.2-38.36) | 77.04 | -0.56 (-1.14-0.02) |
