# Supplementary material for: A comparative analysis of the burden, trends and inequalities of tracheal, bronchus, and lung cancer in India from 2000 to 2021: A systematic analysis for the Global Burden of Disease study 2021
Source: PLoS One. 2025 May 7;20(5):e0322646. doi: 10.1371/journal.pone.0322646 (PMC12058026; doi:10.1371/journal.pone.0322646)
Supplement: S4 Table — (DOCX) [file pone.0322646.s004.docx]

S4 Table. The burden of TBL cancer DALYs associated with tobacco exposure among female in India in 2000 and 2021 and the temporal trend from 2000 to 2021

| female | tobacco | | | | | | smoking | | | | | | secondhand smoke | | | | | |
| --- | --- | --- | --- | --- | --- | --- | --- | --- | --- | --- | --- | --- | --- | --- | --- | --- | --- | --- |
|  | 2000 | | 2021 | | 2000-2021 | | 2000 | | 2021 | | 2000-2021 | | 2000 | | 2021 | | 2000-2021 | |
|  | DALYs case  (95% UI） | ASDR  (95% UI） | DALYs case  (95% UI） | ASDR  (95% UI） | Relative difference(%) | AAPC  (95% CI） | DALYs case  (95% UI） | ASDR  (95% UI） | DALYs case  (95% UI） | ASDR  (95% UI） | Relative difference(%) | AAPC  (95% CI） | DALYs case  (95% UI） | ASDR  (95% UI） | DALYs case  (95% UI） | ASDR  (95% UI） | Relative difference(%) | AAPC  (95% UI） |
| India | 44348.93 (29050-60595) | 13.75 (9.13-18.55) | 99807 (63976-143507) | 15.82 (10.25-22.68) | 125.05 | 0.62 (0.41-0.84) | 32412 (25554-41282) | 10.32 (8.13-13.16) | 74576 (54935-97707) | 11.96 (8.81-15.68) | 130.09 | 0.7 (0.4-1) | 14106 (1874-26765) | 4.12 (0.55-7.84) | 29086 (4040-59621) | 4.48 (0.63-9.22) | 106.19 | 0.35 (0.14-0.56) |
| Andhra Pradesh | 1875  (1116-2933) | 10.99 (6.59-16.98) | 3655 (2057-5582) | 11.7 (6.54-17.83) | 94.96 | 0.28 (-0.11-0.68) | 1597 (972-2462) | 9.42 (5.82-14.51) | 3139 (1925-4726) | 10.04 (6.14-15.12) | 96.6 | 0.28 (-0.16-0.72) | 369 (38-847) | 2.1 (0.22-4.81) | 648 (75-1536) | 2.08 (0.24-4.92) | 75.33 | -0.04 (-0.33-0.25) |
| Assam | 1557 (1025-2206) | 23.95 (16.16-33.56) | 3034 (1685-4895) | 23.37 (13.29-36.87) | 94.92 | -0.11 (-0.35-0.12) | 1248 (882-1687) | 19.82 (14.19-26.74) | 2216 (1416-3308) | 17.56 (11.13-25.96) | 77.51 | -0.51 (-0.91--0.11) | 375 (47-865) | 5.19 (0.66-11.81) | 951 ((129-2231) | 6.86 (0.93-16.16) | 153.64 | 1.33 (0.85-1.81) |
| Bihar | 1667  (1164-2326) | 7.54 (5.3-10.42) | 3415 (2394-4942) | 8.17 (5.74-11.86) | 104.92 | 0.34 (-0.02-0.7) | 1558 (1109-2149) | 7.11 (5.07-9.68) | 3202 (2274-4634) | 7.7 (5.49-11.08) | 105.6 | 0.34 (-0.03-0.71) | 153 (18-317) | 0.63 (0.07-1.32) | 275 (30-642) | 0.62 (0.07-1.45) | 79.47 | -0.09 (-0.37-0.19) |
| Chhattisgarh | 308 (168-493) | 4.52 (2.49-7.15) | 720 (379-1183) | 5.61 (2.89-9.25) | 133.54 | 1.16 (0.25-2.08) | 236 (135-369) | 3.5 (1.99-5.31) | 529 (316-856) | 4.2 (2.45-6.84) | 124.59 | 0.88 (0.03-1.74) | 80 (9-167) | 1.13 (0.13-2.36) | 204 (23-443) | 1.51 (0.17-3.27) | 155.22 | 1.47 (0.81-2.14) |
| Delhi | 843 (528-1300) | 25.53 (16.1-38.71) | 1586 (935-2482) | 17.86 (10.49-28.04) | 88.21 | -1.73 (-2.22--1.23) | 611 (420-864) | 19.04 (12.97-27.5) | 1228 (812-1801) | 13.85 (9.2-20.35) | 100.98 | -1.61 (-2.02--1.19) | 278 (26-623) | 7.92 (0.74-17.79) | 407 (41-945) | 4.56 (0.46-10.46) | 46.69 | -2.66 (-3.36--1.95) |
| Goa | 43 (24-66) | 7.89 (4.35-12.16) | 83 (47-135) | 7.49 (4.25-12.23) | 93.78 | -0.25 (-0.6-0.1) | 36 (21-58) | 6.81 (3.84-10.83) | 71 (42-117) | 6.41 (3.71-10.58) | 96.14 | -0.3 (-0.85-0.26) | 7 (1-16) | 1.22 (0.14-2.67) | 13 (1-30) | 1.18 (0.13-2.74) | 78.08 | -0.18 (-0.69-0.34) |
| Gujarat | 1283 (761-1931) | 8.49 (5.22-12.53) | 4171 (2269-6589) | 12.7 (7.14-19.99) | 224.98 | 2.25 (1.6-2.9) | 869 (590-1218) | 6.1 (4.16-8.48) | 2774 (1804-3958) | 8.66 (5.64-12.28) | 219.01 | 1.9 (0.63-3.18) | 469 (51-981) | 2.77 (0.3-5.77) | 1563 (171-3464) | 4.56 (0.5-10.12) | 233.18 | 2.63 (2.3-2.96) |
| Haryana | 1309 (878-1870) | 20.75 (14.02-29.76) | 3692 (2197-5684) | 28.51 (17.1-43.82) | 182.04 | 1.56 (0.89-2.24) | 986 (710-1406) | 16.04 (11.45-22.92) | 2856 (1782-4232) | 22.31 (14.1-32.83) | 189.79 | 1.64 (0.9-2.4) | 411 (46-838) | 6.15 (0.69-12.6) | 1041 (119-356) | 7.81 (0.89-17.66) | 153.04 | 1.16 (0.77-1.55) |
| Himachal Pradesh | 288 (187-412) | 13.23 (8.76-19.02) | 599 (371-901) | 13.5 (8.41-20.26) | 107.98 | 0.25 (-1.2-1.72) | 229 (156-328) | 10.72 (7.33-15.23) | 481 (322-697) | 10.89 (7.31-15.71) | 110.22 | 0.25 (-1.37-1.89) | 71 (38-149) | 3.08 (0.35-6.44) | 141 (16-314) | 3.14 (0.36-6.98) | 98.18 | 0.18 (-0.82-1.19) |
| Jharkhand | 298 (176-460) | 4.2 (2.42-6.61) | 677 (377-1110) | 4.64 (2.66-7.55) | 127.15 | 0.33 (-1.21-1.89) | 248 (144-384) | 3.57 (2.09-5.57) | 562 (336-903) | 3.89 (2.31-6.32) | 127.04 | 0.26 (-1.33-1.88) | 55 (7-111) | 0.69 (0.09-1.39) | 124 (14-263) | 0.82 (0.1-1.73) | 126.69 | 0.63 (-0.91-2.19) |
| Karnataka | 1774 (956-2796) | 9.42 (5.17-14.69) | 3989 (1915-6700) | 11.5 (5.54-19.29) | 124.78 | 0.98 (0.73-1.23) | 1114 (710-1618) | 6.06 (3.85-8.74) | 2530 (1409-3839) | 7.35 (4.11-11.14) | 127.15 | 0.9 (0.48-1.33) | 731 (95-497) | 3.74 (0.48-7.65) | 1566 (184-3646) | 4.46 (0.53-10.41) | 114.29 | 0.89 (0.64-1.14) |
| Kerala | 2315 (1281-3601) | 15.54 (8.72-24.29) | 3219 (1653-5128) | 12.03 (6.1-19.12) | 39.08 | -1.23 (-1.62--0.84) | 1503 (933-2258) | 10.12 (6.34-15.36) | 2238 (1250-3534) | 8.26 (4.7-12.87) | 48.91 | -0.99 (-1.56--0.41) | 910 (104-1963) | 6.09 (0.71-3.09) | 1051 (110-2308) | 4.03 (0.42-8.85) | 15.46 | -1.97 (-2.67--1.27) |
| Madhya Pradesh | 2270 (1117-3706) | 12.77 (6.43-20.92) | 4173 (2106-7392) | 11.42 (5.74-19.92) | 83.83 | -0.52 (-0.91--0.13) | 1322 (859-1917) | 7.71 (5.01-11.13) | 2527 (1494-4018) | 7.04 (4.19-11.08) | 91.17 | -0.27 (-0.72-0.19) | 1047 (130-2388) | 5.65 (0.69-12.96) | 1762 (186-4213) | 4.71 (0.5-11.17) | 68.29 | -0.82 (-1.24--0.41) |
| Maharashtra | 2930 (1570-4619) | 8.56 (4.72-13.26) | 5456 (2874-9186) | 8.47 (4.47-14.2) | 86.2 | -0.09 (-0.46-0.28) | 1904 (1197-2856) | 5.64 (3.61-8.44) | 3715 (2219-6070) | 5.78 (3.47-9.4) | 95.11 | 0.1 (-0.27-0.48) | 1134 (146-2371) | 3.24 (0.42-6.74) | 1874 (231-4313) | 2.9 (0.36-6.68) | 65.31 | -0.6 (-1.36-0.15) |
| Manipur | 629 (393-900) | 104.44 (66.26-149.21) | 1473 (937-2163) | 103.27 (67.04-150.15) | 134.11 | -0.08 (-0.57-0.41) | 580 (366-817) | 97.32 (61.06-136.75) | 1346 (852-1939) | 95.45 (61.77-135.53) | 132.06 | -0.12 (-0.61-0.37) | 78 (9-66) | 11.83 (1.28-25.22) | 182 (22-409) | 11.62 (1.41-25.62) | 133.83 | 0.01 (-0.16-0.17) |
| Meghalaya | 176 (92-281) | 37.14 (19.16-59.41) | 417 (236-667) | 39.18 (22.3-61.3) | 137.39 | 0.22 (-0.4-0.84) | 130 (77-195) | 28.5 (16.73-42.97) | 292 (180-427) | 28.06 (17.35-41.62) | 123.63 | -0.12 (-0.82-0.58) | 57 (6-137) | 11.28 (1.19-27.09) | 151 (20-338) | 13.57 (1.84-30.38) | 163.15 | 0.87 (0.33-1.42) |
| Mizoram | 606 (400-881) | 283.29 (186.82-411.68) | 1659 (1045-2528) | 333.84 (213.88-500.74) | 174.06 | 0.61 (-0.03-1.25) | 545 (367-789) | 257.79 (172.9-376.59) | 1476 (954-2192) | 299.62 (198-436.28) | 171.03 | 0.67 (0.07-1.26) | 104 (10-223) | 45.93 (4.48-98.94) | 293 (31-651) | 56.56 (6.06-125.1) | 182.72 | 1 (0.85-1.15) |
| Nagaland | 49 (24-82) | 12.68 (6.22-21.38) | 71 (34-119) | 11.62 (5.64-19.06) | 45.73 | -0.38 (-0.88-0.13) | 31 (19-48) | 8.37 (5.01-12.65) | 46 (27-74) | 7.66 (4.59-12.39) | 48.48 | -0.38 (-0.88-0.12) | 19 (2-45) | 4.77 (0.49-11.23) | 27 (4-59) | 4.23 (0.59-9.45) | 36.9 | -0.55 (-0.77--0.33) |
| Odisha | 666 (364-1053) | 5.01 (2.77-7.83) | 1542 (790-2550) | 6.27 (3.23-10.28) | 131.57 | 1.14 (0.03-2.26) | 452 (287-697) | 3.44 (2.17-5.22) | 1098 (653-1823) | 4.46 (2.67-7.34) | 142.8 | 1.55 (1.32-1.78) | 233 (29-526) | 1.72 (0.21-3.9) | 478 (54-1105) | 1.95 (0.22-4.48) | 105.67 | 0.63 (-0.04-1.3) |
| Other Union Territories | 54 (23-92) | 7.05 (3.23-11.86) | 164 (75-283) | 8.88 (4.09-15.26) | 205.11 | 1.1 (0.85-1.35) | 28 (16-46) | 3.96 (2.35-6.41) | 92 (50-154) | 5.12 (2.76-8.55) | 221.82 | 1.17 (0.89-1.45) | 27 (3-61) | 3.27 (0.36-7.3) | 76 (9-175) | 3.97 (0.47-9.22) | 185.6 | 0.87 (0.58-1.18) |
| Punjab | 548 (280-871) | 6.47 (3.38-10.27) | 889 (444-1530) | 5.27 (2.64-9.03) | 62.41 | -0.96 (-1.39--0.52) | 333 (200-510) | 4.04 (2.47-6.08) | 551 (304-884) | 3.31 (1.88-5.29) | 65.33 | -0.93 (-1.4--0.46) | 227 (29-487) | 2.57 (0.33-5.51) | 353 (43-823) | 2.05 (0.25-4.81) | 55.86 | -1.07 (-1.53--0.61) |
| Rajasthan | 3018 (1972-4286) | 18.29 (11.93-25.87) | 6594 (3823-10538) | 20.58 (12.09-32.87) | 118.46 | 0.56 (0.27-0.85) | 2421 (1725-3259) | 14.95 (10.48-20.14) | 5107 (3251-7676) | 16.19 (10.41-24.29) | 110.91 | 0.38 (0.02-0.74) | 822 (108-1668) | 4.72 (0.62-9.66) | 1809 (209-4225) | 5.41 (0.63-12.58) | 120.13 | 0.65 (0.47-0.84) |
| Sikkim | 81 (53-119) | 72.3 (47.76-105.84) | 187 (118-301) | 71.04 (45.02-113.33) | 130.62 | -0.15 (-0.55-0.26) | 73 (49-106) | 65.83 (44.63-96.02) | 167 (107-253) | 63.87 (40.67-97.75) | 128.47 | -0.2 (-0.62-0.22) | 12 (1-26) | 9.65 (1.02-20.95) | 26 (3-65) | 9.49 (1.17-23.59) | 126.12 | -0.12 (-0.54-0.29) |
| Tamil Nadu | 1567 (712-2726) | 6.36 (2.94-11.05) | 2388 (1170-4103) | 5.1 (2.52-8.74) | 52.46 | -1.06 (-1.4--0.72) | 765 (467-1207) | 3.21 (2.02-5.01) | 1355 (835-2166) | 2.93 (1.81-4.62) | 77.24 | -0.42 (-1.01-0.18) | 848 (98-1862) | 3.34 (0.39-7.38) | 1077 (127-2394) | 2.26 (0.27-5) | 26.96 | -1.81 (-2.19--1.43) |
| Telangana | 784 (451-1248) | 7.99 (4.65-12.52) | 2497 (1453-3729) | 13.34 (7.72-20.23) | 218.34 | 2.44 (2.17-2.71) | 658 (381-1049) | 6.86 (4.11-0.78) | 2112 (1275-3112) | 11.39 (6.86-16.52) | 220.91 | 2.41 (2.14-2.68) | 145 (13-343) | 1.33 (0.12-3.14) | 454 (56-1078) | 2.32 (0.28-5.49) | 212.95 | 2.65 (2.32-2.98) |
| Tripura | 294 (185-459) | 31.41 (20.03-48.82) | 551 (333-819) | 30.04 (18.21-44.56) | 87.52 | -0.15 (-0.63-0.34) | 257 (169-382) | 27.84 (18.3-40.9) | 481 (301-709) | 26.42 (16.78-38.87) | 87.11 | -0.19 (-0.66-0.29) | 57 (6-133) | 5.73 (0.61-3.36) | 96 (13-209) | 5.06 (0.65-10.96) | 70.11 | -0.42 (-1.02-0.19) |
| Uttar Pradesh | 8842 (5509-13041) | 19.57 (12.35-28.36) | 22879 (14095-36015) | 25.99 (16.22-40.28) | 158.75 | 1.44 (0.66-2.23) | 6853 (4769-9239) | 15.66 (10.9-21.09) | 18191 (12237-26593) | 21.05 (14.19-30.53) | 165.46 | 1.4 (0.7-2.1) | 2464 (271-5524) | 4.99 (0.55-11.22) | 5628 (693-14401) | 6.03 (0.74-15.52) | 128.43 | 0.93 (0.51-1.35) |
| Uttarakhand | 1258 (800-1825) | 49.35 (31.62-71.43) | 4158 (2606-6135) | 77.82 (49.62-113.75) | 230.54 | 2.21 (1.77-2.65) | 1017 (683-1427) | 40.96 (27.53-57.09) | 3486  (2317-4936) | 66.01 (44.62-92.55) | 242.78 | 2.32 (1.84-2.8) | 311 (29-663) | 11.2 (1.08-23.91) | 883 (97-1937) | 15.77 (1.72-34.63) | 183.51 | 1.65 (1.35-1.96) |
| West Bengal | 5315 (2851-7930) | 22.29 (12.47-32.98) | 11940 (6297-19492) | 25.29 (13.72-40.7) | 124.64 | 0.62 (0.27-0.97) | 3375 (2366-4616) | 14.94 (10.5-20.27) | 7513 (4922-11369) | 16.48 (10.9-24.52) | 122.61 | 0.41 (0.04-0.78) | 2239 (1302-4527) | 8.68 (1.15-17.46) | 4974 (556-10175) | 10.01 (1.12-20.37) | 122.16 | 0.58 (0.21-0.95) |
