# Supplementary figures and images for: A comparative analysis of the burden, trends and inequalities of tracheal, bronchus, and lung cancer in India from 2000 to 2021: A systematic analysis for the Global Burden of Disease study 2021
Source: PLoS One. 2025 May 7;20(5):e0322646. doi: 10.1371/journal.pone.0322646 (PMC12058026; doi:10.1371/journal.pone.0322646)

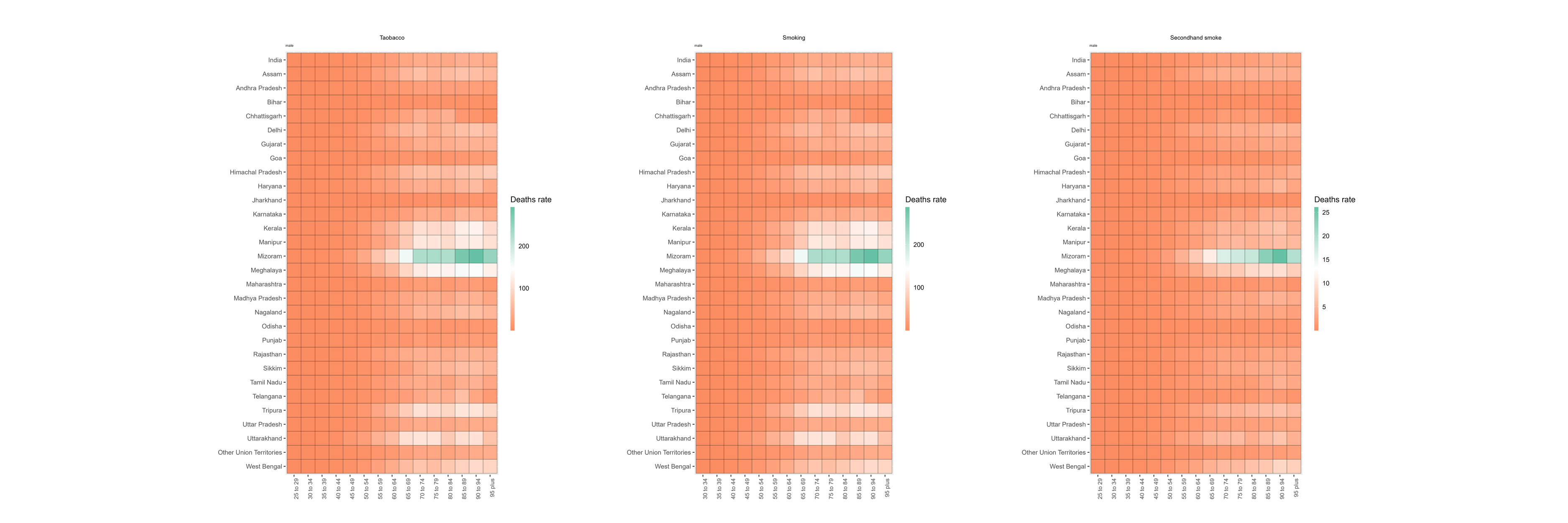

Supplement: S1 Fig — (TIF) [file pone.0322646.s005.tif]

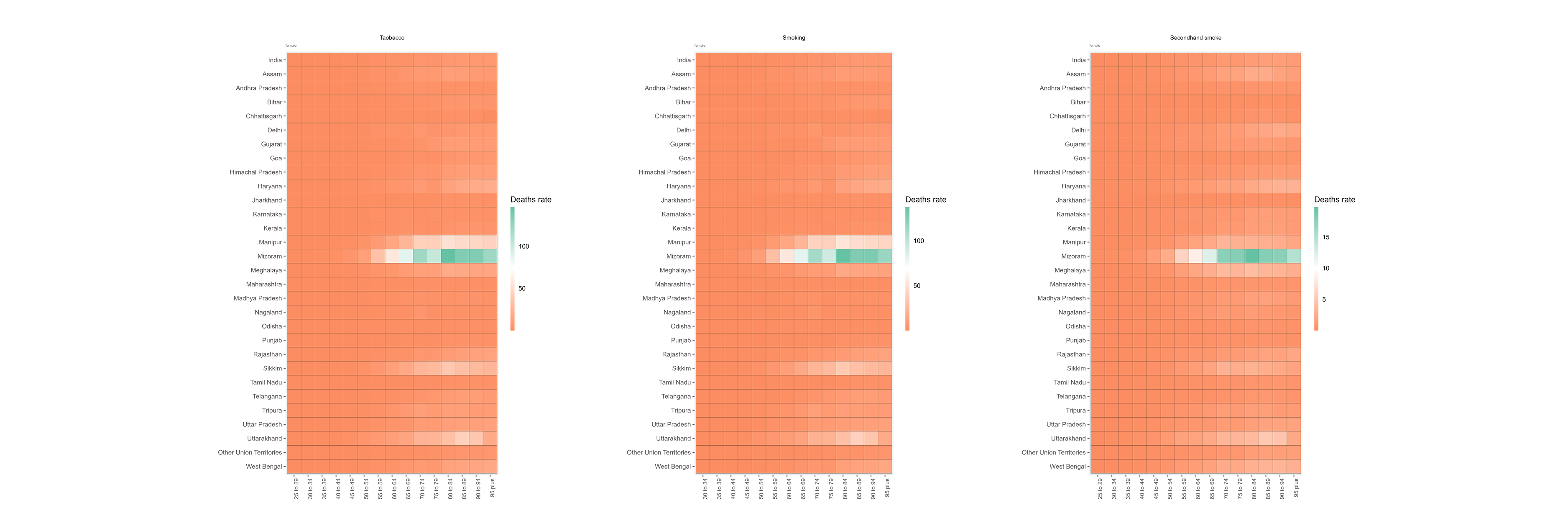

Supplement: S2 Fig — (TIF) [file pone.0322646.s006.tif]

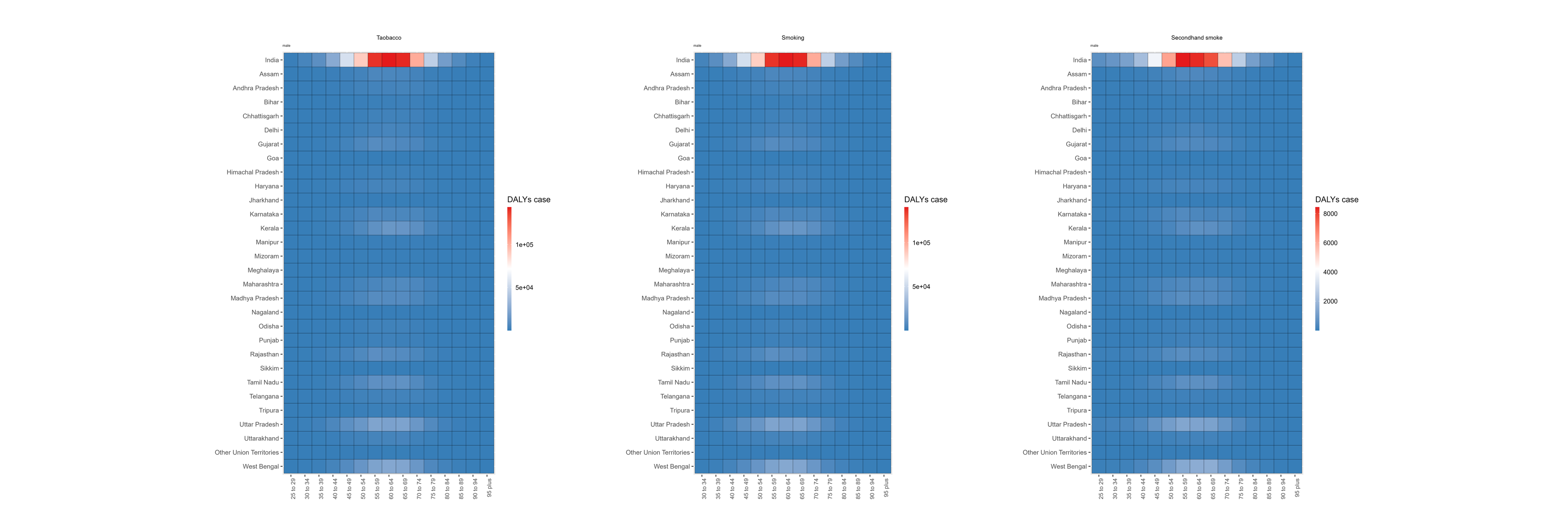

Supplement: S3 Fig — (TIF) [file pone.0322646.s007.tif]

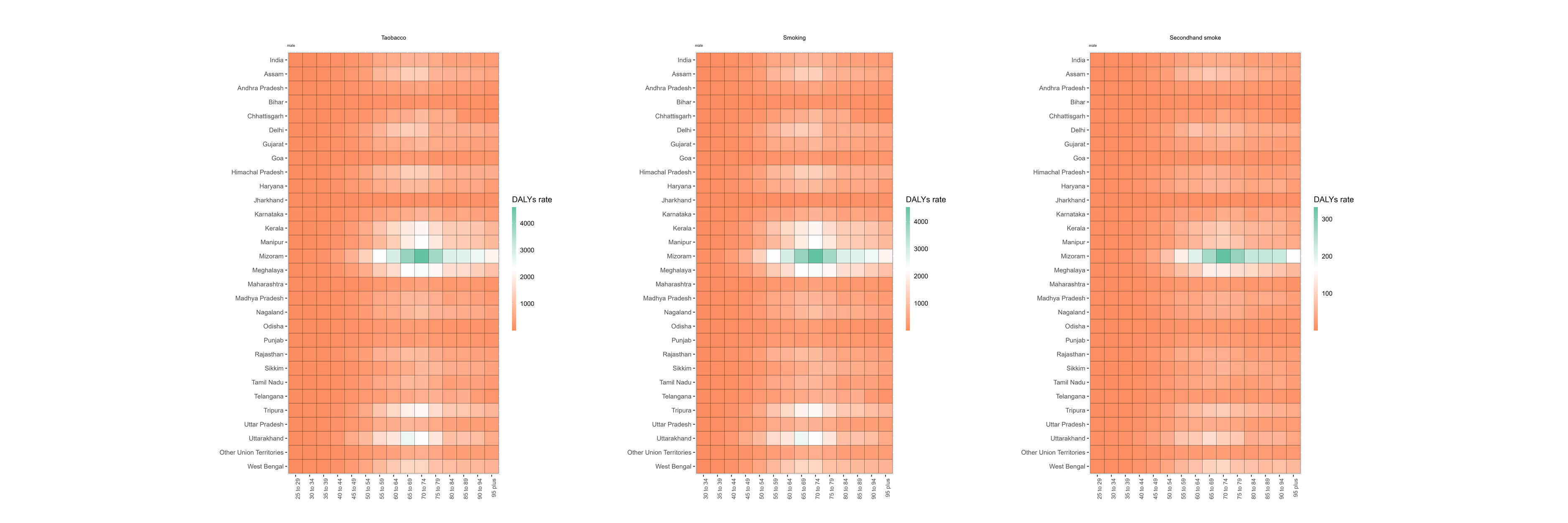

Supplement: S4 Fig — (TIF) [file pone.0322646.s008.tif]

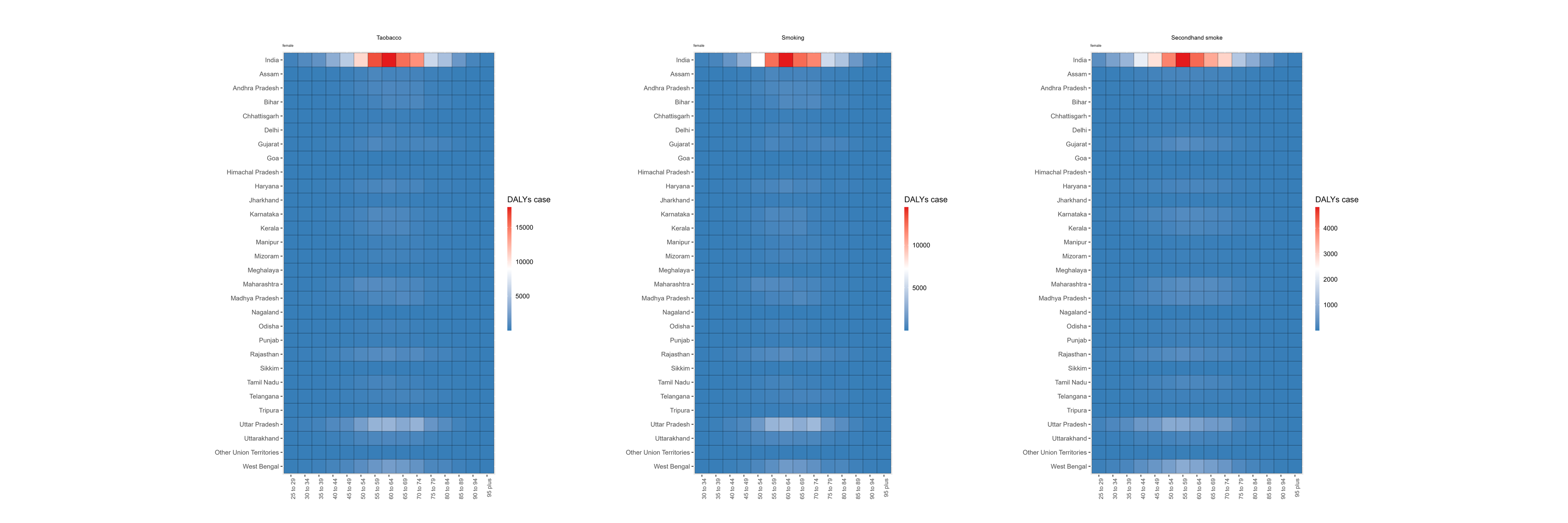

Supplement: S5 Fig — (TIF) [file pone.0322646.s009.tif]

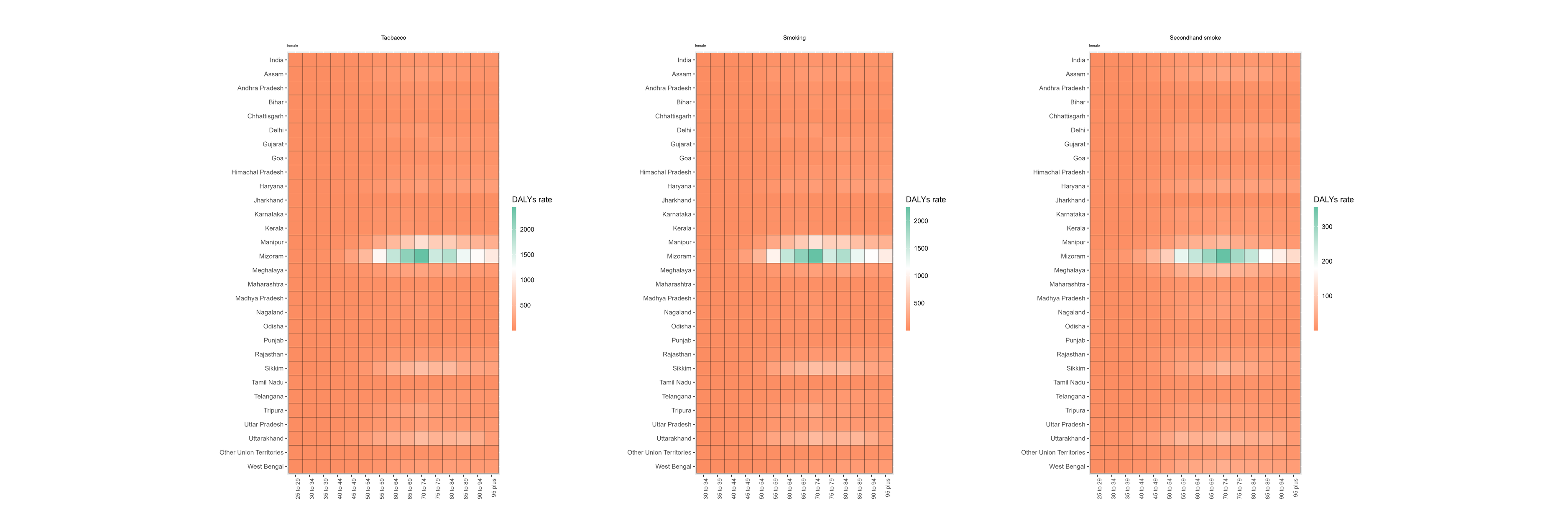

Supplement: S6 Fig — (TIF) [file pone.0322646.s010.tif]

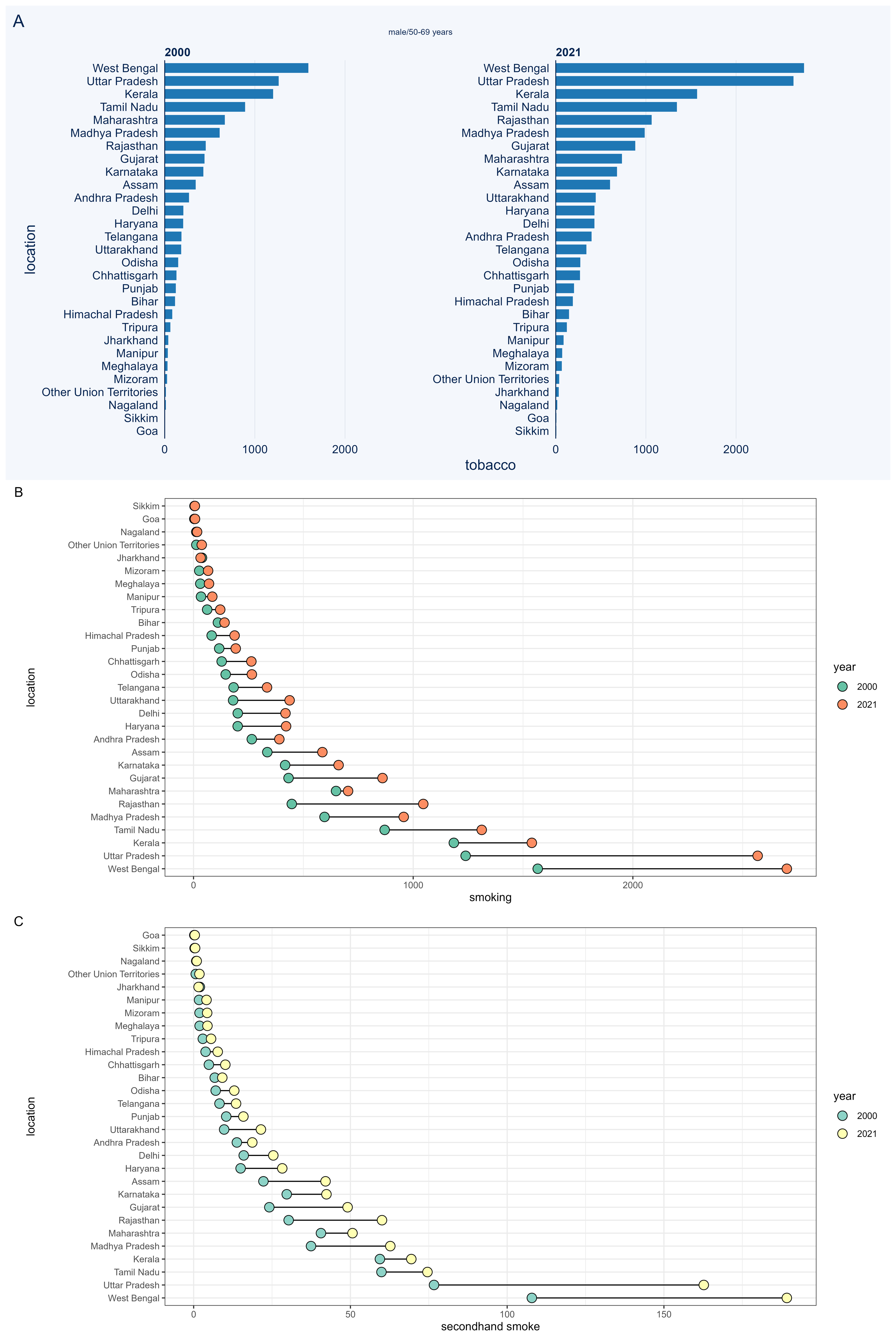

Supplement: S7 Fig — (a) tobacco, (b) smoking, (c) second-hand smoke. (TIF) [file pone.0322646.s011.tif]

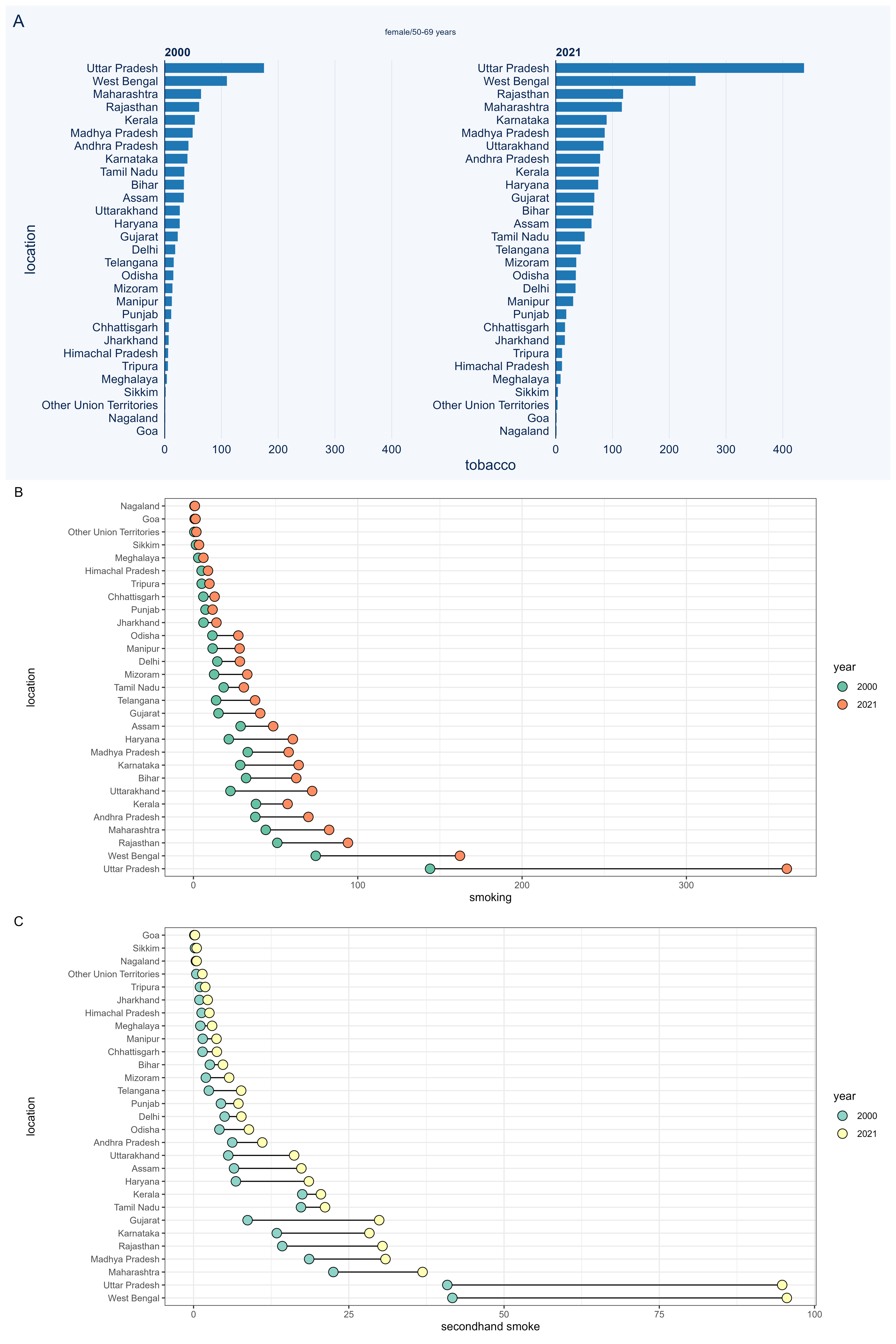

Supplement: S8 Fig — (a) tobacco, (b) smoking, (c) second-hand smoke. (TIF) [file pone.0322646.s012.tif]

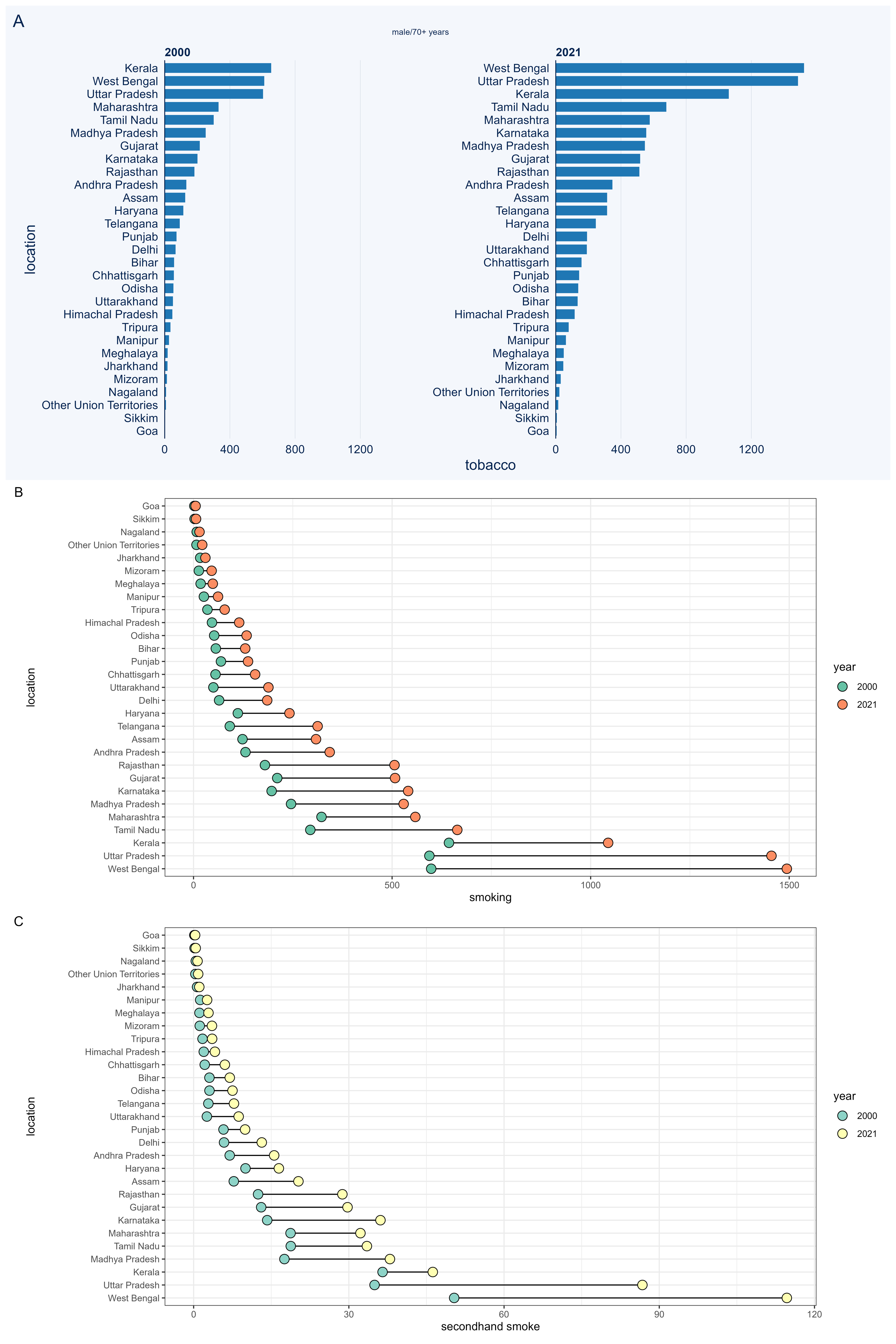

Supplement: S9 Fig — (a) tobacco, (b) smoking, (c) second-hand smoke. (TIF) [file pone.0322646.s013.tif]

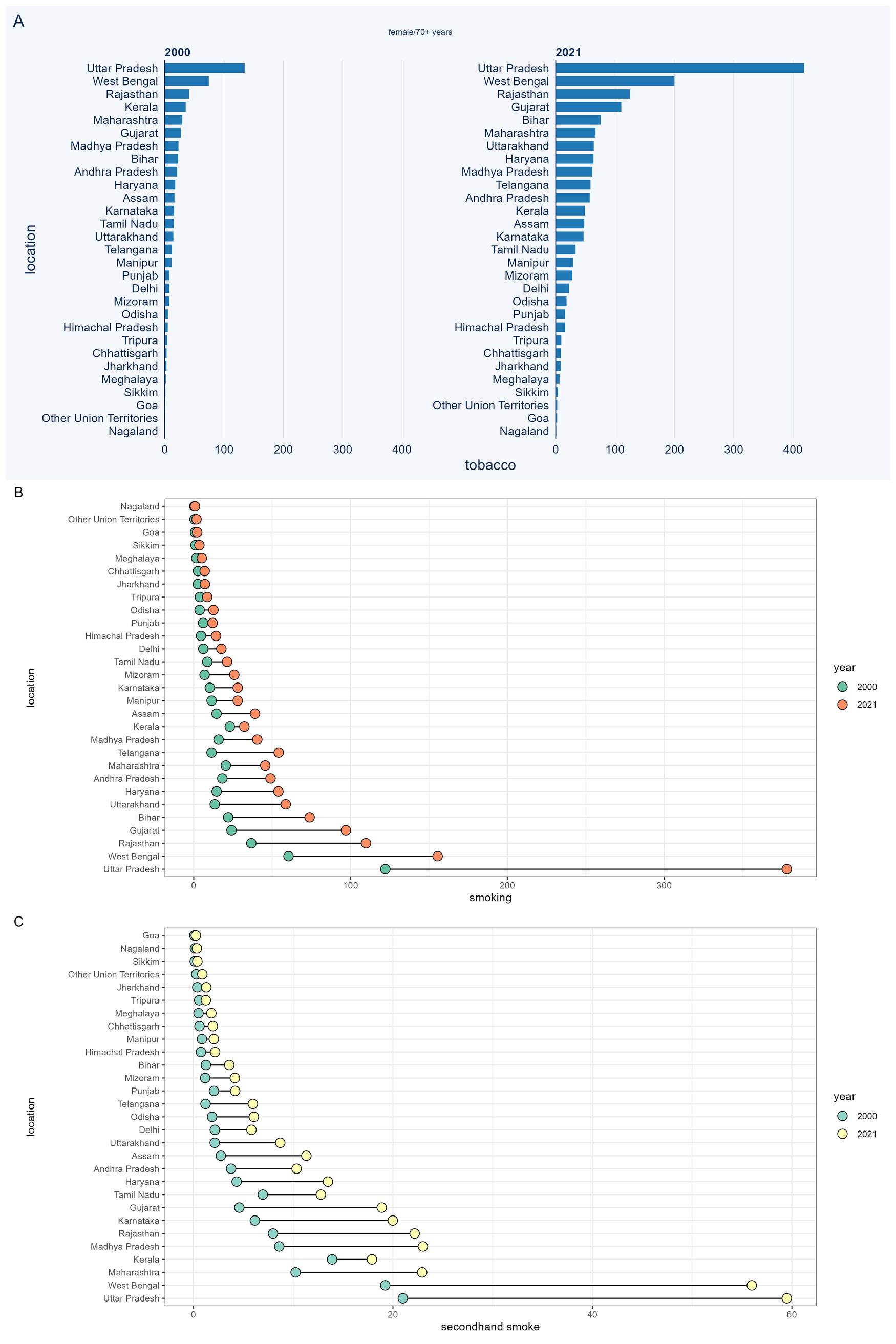

Supplement: S10 Fig — (a) tobacco, (b) smoking, (c) second-hand smoke. (TIF) [file pone.0322646.s014.tif]

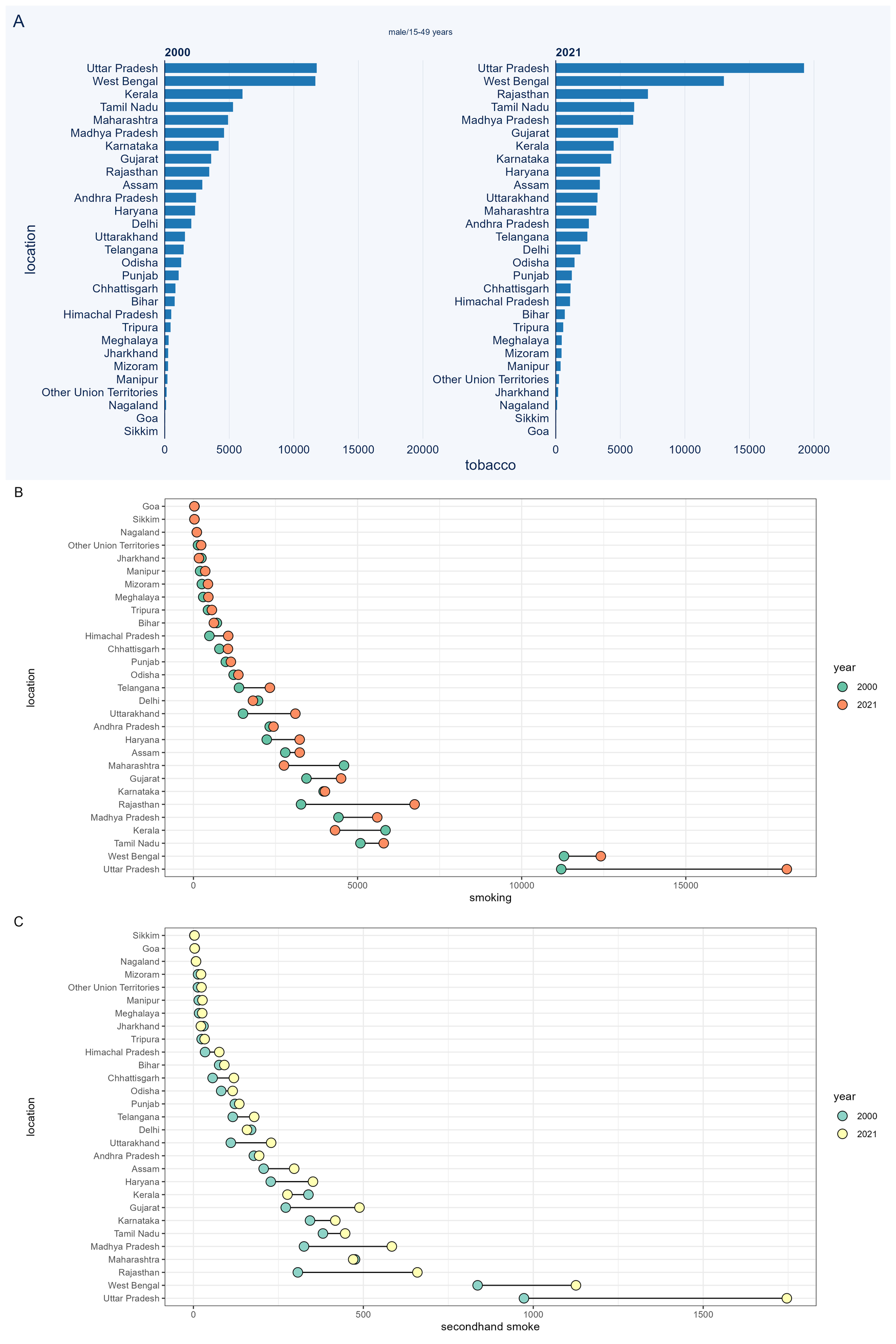

Supplement: S11 Fig — (a) tobacco, (b) smoking, (c) second-hand smoke. (TIF) [file pone.0322646.s015.tif]

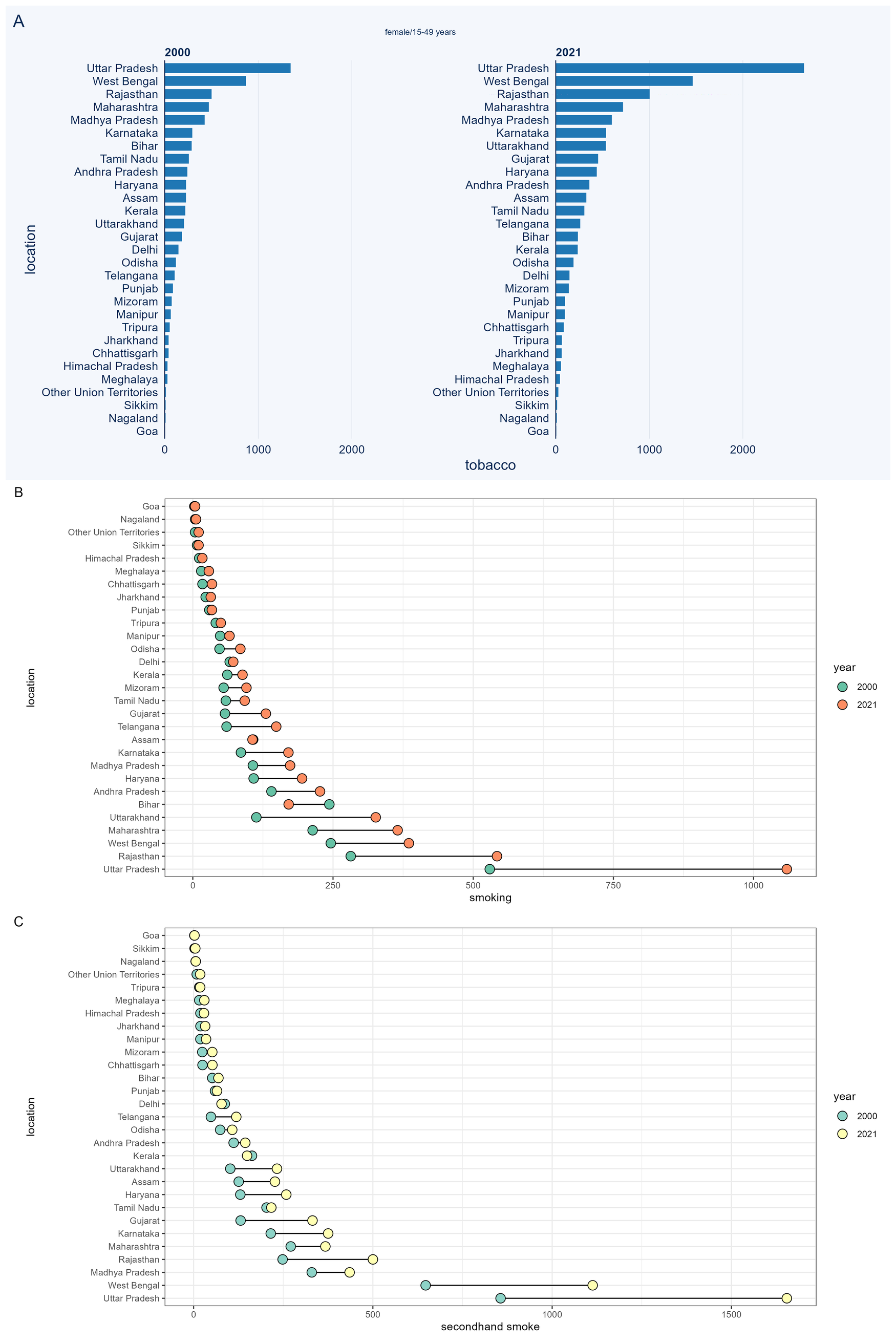

Supplement: S12 Fig — (a) tobacco, (b) smoking, (c) second-hand smoke. (TIF) [file pone.0322646.s016.tif]

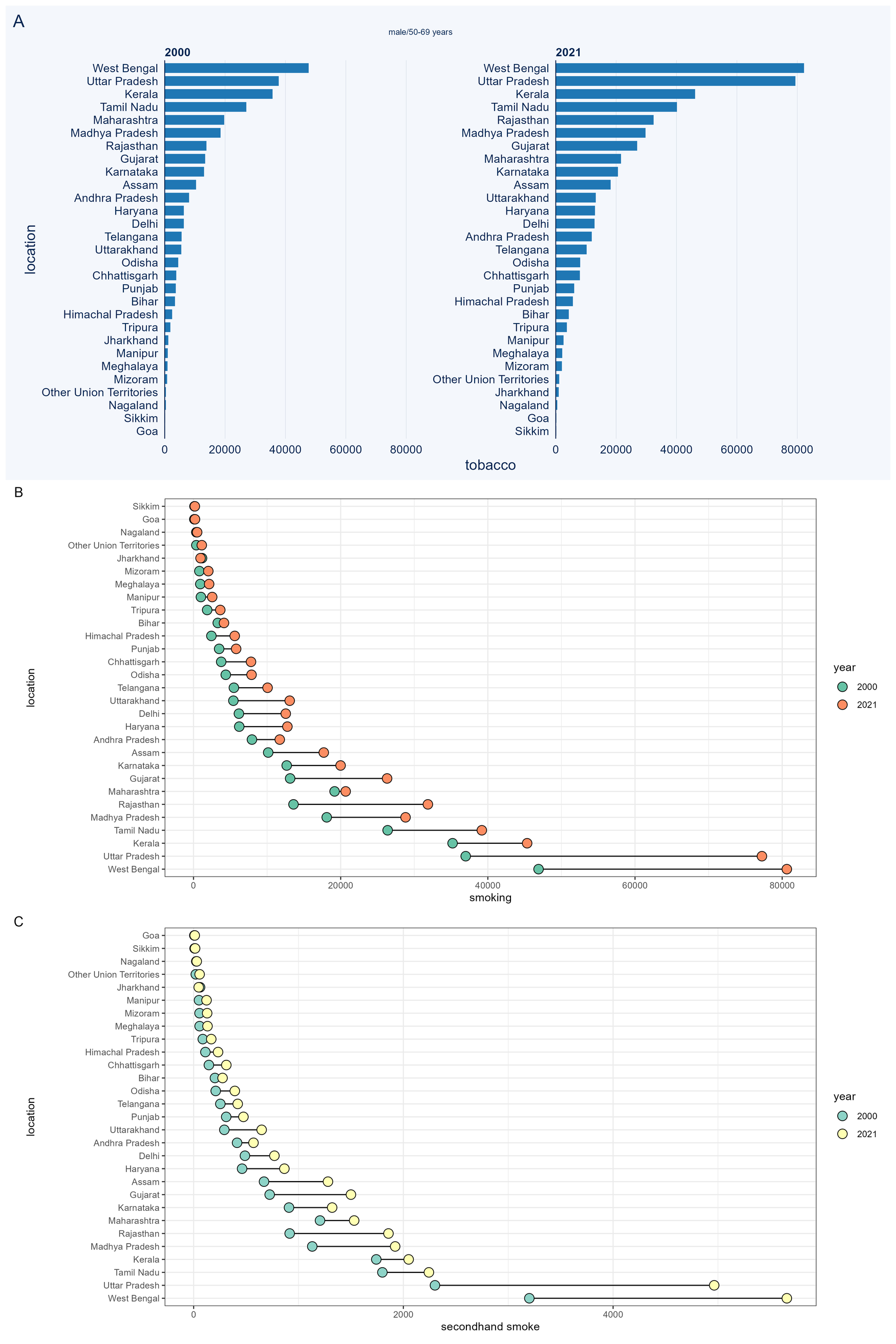

Supplement: S13 Fig — (a) tobacco, (b) smoking, (c) second-hand smoke. (TIF) [file pone.0322646.s017.tif]

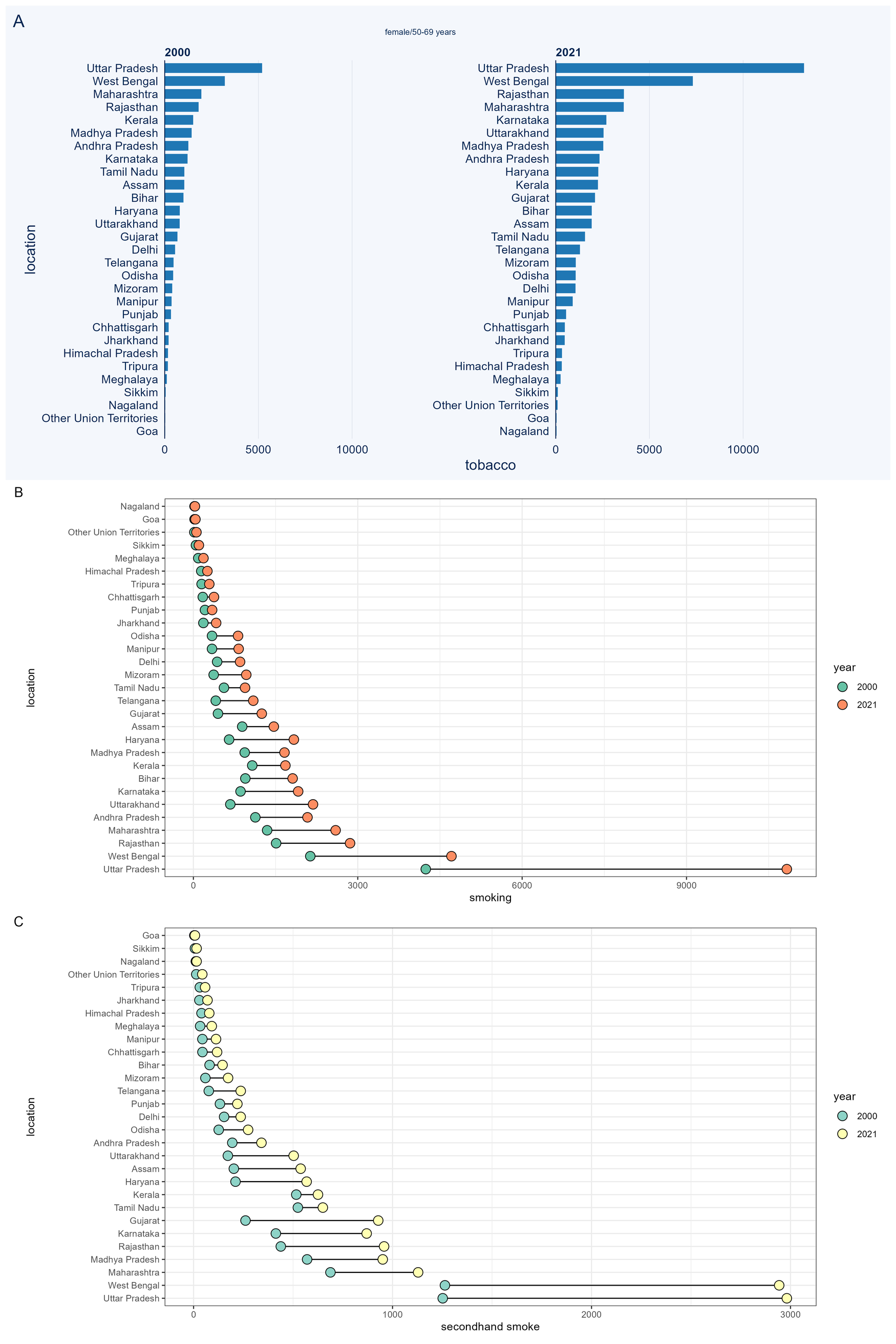

Supplement: S14 Fig — (a) tobacco, (b) smoking, (c) second-hand smoke. (TIF) [file pone.0322646.s018.tif]
